# Nurr1 Modulation Mediates Neuroprotective Effects of Statins

**DOI:** 10.1002/advs.202104640

**Published:** 2022-04-30

**Authors:** Sabine Willems, Julian A. Marschner, Whitney Kilu, Giuseppe Faudone, Romy Busch, Silke Duensing‐Kropp, Jan Heering, Daniel Merk

**Affiliations:** ^1^ Institute of Pharmaceutical Chemistry Goethe University Frankfurt Max‐von‐Laue‐Str. 9 Frankfurt 60438 Germany; ^2^ Department of Pharmacy Ludwig‐Maximilians‐Universität München Butenandtstr. 5‐13 Munich 81377 Germany; ^3^ Fraunhofer Institute for Translational Medicine and Pharmacology ITMP Theodor‐Stern‐Kai 7 Frankfurt 60596 Germany

**Keywords:** Alzheimer's disease, multiple sclerosis, NR4A2, nuclear receptor related‐1, Parkinson's disease

## Abstract

The ligand‐sensing transcription factor Nurr1 emerges as a promising therapeutic target for neurodegenerative pathologies but Nurr1 ligands for functional studies and therapeutic validation are lacking. Here pronounced Nurr1 modulation by statins for which clinically relevant neuroprotective effects are demonstrated, is reported. Several statins directly affect Nurr1 activity in cellular and cell‐free settings with low micromolar to sub‐micromolar potencies. Simvastatin as example exhibits anti‐inflammatory effects in astrocytes, which are abrogated by Nurr1 knockdown. Differential gene expression analysis in native and Nurr1‐silenced cells reveals strong proinflammatory effects of Nurr1 knockdown while simvastatin treatment induces several neuroprotective mechanisms via Nurr1 involving changes in inflammatory, metabolic and cell cycle gene expression. Further in vitro evaluation confirms reduced inflammatory response, improved glucose metabolism, and cell cycle inhibition of simvastatin‐treated neuronal cells. These findings suggest Nurr1 involvement in the well‐documented but mechanistically elusive neuroprotection by statins.

## Graphical abstract

Simvastatin modulates Nurr1 activity and alters the expression of genes involved in inflammation, glucose metabolism, and cell cycle regulation in astrocytes in a Nurr1 dependent fashion. In consequence, simvastatin‐treated astrocytes display a reduced inflammatory response, enhanced glucose consumption, and lower cell cycle activity. Nurr1‐mediated effects may hence importantly contribute to neuroprotection by simvastatin.

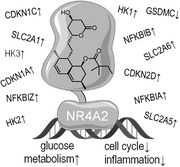

## 1 Introduction

The ligand‐activated transcription factor nuclear receptor related‐1 (Nurr1, NR4A2)^[^ ^1^ ^]^ is a constitutively active orphan nuclear receptor. It is considered as neuroprotective transcriptional regulator and ascribed high therapeutic potential in neurodegenerative diseases. Nurr1 is expressed in several neuronal cell populations with highest levels in dopaminergic neurons and thought to protect neurons against injury.^[^ ^2^ ^]^ Neuronal Nurr1 knockout in mice produced a phenotype resembling Parkinson's disease (PD)^[^ ^2^, ^3^ ^]^ and in the neurotoxin 1‐methyl‐4‐phenyl‐1,2,3,6‐tetrahydropyridine (MPTP)‐induced model of PD in rodents, Nurr1 was downregulated resulting in neuroinflammation and enhanced apoptosis of neuronal cells^[^ ^4^ ^]^ while Nurr1 overexpression in the same model reduced motor impairment and spatial learning deficits.^[^ ^4^ ^]^ In experimental autoimmune encephalomyelitis (EAE), heterozygous Nurr1 knockout mice developed the disease faster than wild‐type mice,^[^ ^5^ ^]^ while enhanced Nurr1 signaling reduced incidence and severity of EAE.^[^ ^6^ ^]^ Neuronal Nurr1 expression was also significantly downregulated in models of Alzheimer's disease (AD) in an age‐dependent fashion^[^ ^7^, ^8^ ^]^ and the transcription factor was shown to protect against AD‐related pathology including Aβ accumulation, neuronal loss, and microglial activation in vivo.^[^ ^8^ ^]^ In line with these observations from rodent models, altered Nurr1 expression has been detected in human PD, AD, and multiple sclerosis (MS) patients^[^ ^4^, ^7^, ^8^, ^9^ ^]^ further highlighting the great neuroprotective potential of Nurr1,^[^ ^10^ ^]^ which may hence be a very attractive therapeutic target to treat neurodegenerative pathologies.

Despite this therapeutic promise, knowledge on Nurr1 function and ligands is still scarce. A few weak Nurr1 modulators have been discovered^[^ ^3^, ^11^, ^12^, ^13^, ^14^, ^15^, ^16^ ^]^ such as the prostaglandins A1 and E1 as potential endogenous ligands.^[^ ^12^ ^]^ The antimalarials amodiaquine (AQ) and chloroquine (CQ) have served as early Nurr1 agonist tools to evaluate Nurr1 activation in neurodegeneration.^[^ ^3^, ^8^, ^17^ ^]^ Therapeutic validation of Nurr1 in neurodegenerative pathologies and beyond, however, requires potent and selective Nurr1 modulators. Aiming to close this gap and expand the collection of Nurr1 ligand scaffolds, we have screened a drug fragment library for Nurr1 modulation in a cellular setting resulting in the discovery of statins as potent Nurr1 modulators. Intrigued by this finding and reports on clinically relevant effects of this drug class in neurodegeneration,^[^ ^18^, ^19^, ^20^ ^]^ we have evaluated the potential involvement of Nurr1 in the neuroprotective actions of statins. Differential gene expression experiments in native and Nurr1‐silenced astrocytes demonstrated several Nurr1‐mediated effects of simvastatin. Among them, reduced inflammation, improved glucose metabolism and energy generation, and cell cycle inhibition upon simvastatin treatment were observed on gene expression level and confirmed in cellular settings. These effects emerge as Nurr1‐mediated neuroprotective mechanisms of simvastatin indicating important contributions of Nurr1 modulation in the pharmacological effects of simvastatin and related drugs in neurodegeneration.

## 2 Results

### 2.1 Fragment Screening Reveals Structurally Diverse Nurr1 Ligands

**Figure 1:**
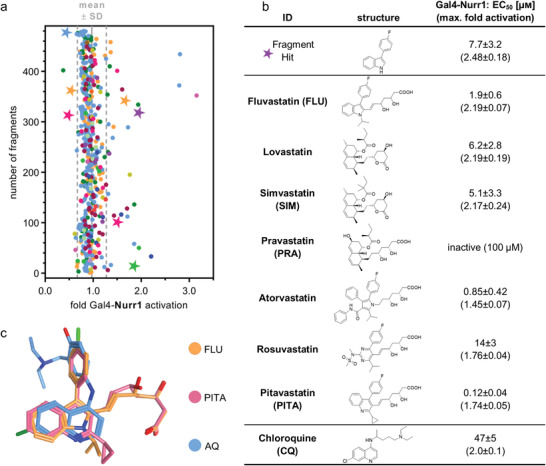
Discovery of statins as Nurr1 modulators and their profiling. a) Primary fragment screening results. Nurr1 modulatory activity of the entire drug fragment library in a Gal4‐Nurr1 hybrid reporter gene assay. Data are the mean reporter activity versus 0.4% DMSO at 100 × 10^−6^ m; *n* = 2. Different colors represent different graph frameworks (see also Figure S1, Supporting Information). Compounds marked with a star relate to the fragment hits validated in control experiments on Gal4‐VP16. Gray lines represent mean ± SD of the entire screening. b) Nurr1 modulatory activity of the fragment screening hit and of the statin class of drugs (vs 0.1% DMSO) in a Gal4‐Nurr1 hybrid reporter gene assay. Data for chloroquine (CQ) from ref. [15]. Data are the mean ± S.E.M.; *n* ≥ 3. c) Multiple alignment of fluvastatin (FLU), pitavastatin (PITA), and amodiaquine (AQ) reveals common structural features with overlap of the indole and quinoline scaffolds as well as the phenyl substituents.

As rapid approach to discover Nurr1 ligands, we have screened a commercially available collection of 480 drug fragments (see Figure S1 of the Supporting Information for details) for Nurr1 modulation in a cellular Gal4‐Nurr1 hybrid reporter gene assay at a single concentration of 100 × 10^−6^ m. Fragments affecting reporter activity ≥ 1.5‐fold (Nurr1 activation) or ≤ 0.6‐fold (Nurr1 repression) were considered for further evaluation (**Figure** **1**; Figure S2a, Supporting Information). Curation for toxicity and PAINs structures, and control experiments for nonspecific effects on reporter activity (using Gal4‐VP16^[^ ^21^, ^22^ ^]^) resulted in a collection of seven Nurr1 ligand fragments with no privileged scaffold for further characterization. Four fragments promoted Nurr1 activity and three fragments acted as inverse Nurr1 agonists (Figures S2 and S3, Supporting Information). 3‐(4‐Fluorophenyl)indole emerged as most active Nurr1 activator fragment (EC_50_ 7.7 × 10^−6^ m, 2.5‐fold eff.). It is contained in the widely used cholesterol‐lowering drug fluvastatin which was an even more potent Nurr1 agonist (EC_50_ 1.9 × 10^−6^ m, 2.2‐fold eff.). Following this remarkable finding, we tested all seven marketed statins (fluvastatin, lovastatin, simvastatin, pravastatin, atorvastatin, rosuvastatin, and pitavastatin) for Nurr1 modulatory activity and observed Nurr1 agonism for all seven drugs except pravastatin (Figure 1b) but with differing potencies. Lovastatin and simvastatin demonstrated similar potencies as fluvastatin while rosuvastatin was less active. Atorvastatin weakly activated Nurr1 (1.5‐fold eff.) with sub‐micromolar potency (EC_50_ 0.85 × 10^−6^ m) and pitavastatin evolved as the most potent Nurr1 agonist amongst statins (EC_50_ 0.12 × 10^−6^ m, 1.7‐fold eff.). Interestingly, statins share structural features with known Nurr1 modulators. Fluvastatin and pitavastatin comprise a similar bicyclic nitrogen‐containing scaffold as AQ,^[^ ^3^ ^]^ CQ^[^ ^3^ ^]^ (Figure 1c), and analogues^[^ ^15^, ^23^ ^]^ which is also contained in the Nurr1 binding dopamine metabolite dihydroxyindole^[^ ^13^, ^24^ ^]^ and indole‐based Nurr1 modulators.^[^ ^25^ ^]^ Atorvastatin and rosuvastatin share substructures with certain non‐steroidal anti‐inflammatory drugs (NSAIDs), which were found to bind Nurr1, too.^[^ ^14^ ^]^ Lovastatin and simvastatin, by contrast, lack structural features that have been previously associated with Nurr1 modulation. Compared to the previously identified ligands, statins exhibit remarkably higher potencies on Nurr1 (Figure 1b). A selectivity screen over other lipid sensing nuclear receptors revealed no further activities of simvastatin and fluvastatin (Figure S4h, Supporting Information).

### 2.2 Statins Modulate Nurr1 Activity in Cellular and Cell‐Free Settings

**Figure 2:**
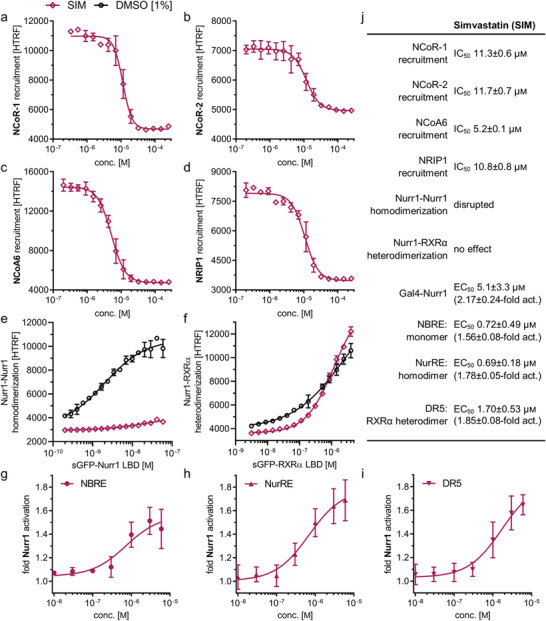
Profiling of simvastatin (SIM) as Nurr1 modulator. a–f) Effects of SIM on coregulator interactions and dimerization of Nurr1 in HTRF assays. SIM displaced a) NCoR‐1, b) NCoR‐2, c) NCoA6, and d) NRIP1 from the Nurr1 LBD in a dose‐dependent fashion and 30 × 10^−6^ m SIM decreased e) homodimerization of Nurr1 f) without affecting Nurr1‐RXRα heterodimerization. Data are the mean ± SD; *N* = 3. g–i) Profiling of SIM in human full‐length Nurr1 reporter gene assays for the Nurr1 response elements NBRE (g, Nurr1 monomer), NurRE (h, Nurr1 homodimer), and DR5 (i, Nurr1‐RXRα heterodimer). Data are the mean ± S.E.M., *n* ≥ 3. j) Summarized activities of Nurr1 modulator SIM in cell‐free and cellular experiments.

To further characterize the intriguing Nurr1 agonism of statins, we selected fluvastatin for its high Nurr1 activation efficacy and the most widely used statin simvastatin as representative compounds. Evidence from clinical use especially points to neuroprotective potential of simvastatin^[^ ^18^, ^19^, ^20^ ^]^ providing additional motivation to study the molecular mechanisms underlying its effects. Moreover, simvastatin lacks a chromophore and was therefore best suited for homogenous time‐resolved fluorescence resonance energy transfer (HTRF)‐based assays. To obtain mechanistic insights in Nurr1 modulation by statins, we evaluated modulation of Nurr1 interactions with coregulators by statins in cell‐free HTRF based systems. We have previously discovered ligand‐sensitive interaction of Nurr1 with nuclear receptor corepressors (NCoR) 1 and 2, nuclear receptor interacting protein 1 (NRIP1) and nuclear receptor coactivator 6 (NCoA6).^[^ ^14^ ^]^ Fluvastatin and simvastatin caused a concentration‐dependent displacement of all four coregulators (**Figure** **2**a–d; Figure S4, Supporting Information). In addition, since Nurr1 can act as monomer, homodimer, and RXR‐heterodimer on different DNA response elements, its activity also depends on its dimerization state.^[^ ^14^, ^26^ ^]^ Fluvastatin and simvastatin did not alter heterodimerization of Nurr1 with RXRα but robustly inhibited Nurr1 homodimerization (Figure 2e,f; Figure S4, Supporting Information). The HTRF assays revealed higher potency of simvastatin compared to fluvastatin prompting us to perform further experiments with simvastatin. Next, we characterized the ability of simvastatin to modulate full‐length human Nurr1 on the human monomer (NGFI‐B response element, NBRE), homodimer (Nur‐response element, NurRE), and heterodimer (direct repeat 5, DR5) response elements.^[^ ^26^ ^]^ Simvastatin activated the full‐length human Nurr1 on all three response elements with low micromolar to sub‐micromolar potencies (Figure 2g–j). Despite disrupting Nurr1 homodimerization, simvastatin also activated the homodimer response element NurRE. As NurRE naturally also contains a Nurr1 monomer binding site,^[^ ^14^, ^27^ ^]^ this finding is not surprising, however.

### 2.3 Statins Block the Inflammatory Response of Astrocytes

**Figure 3:**
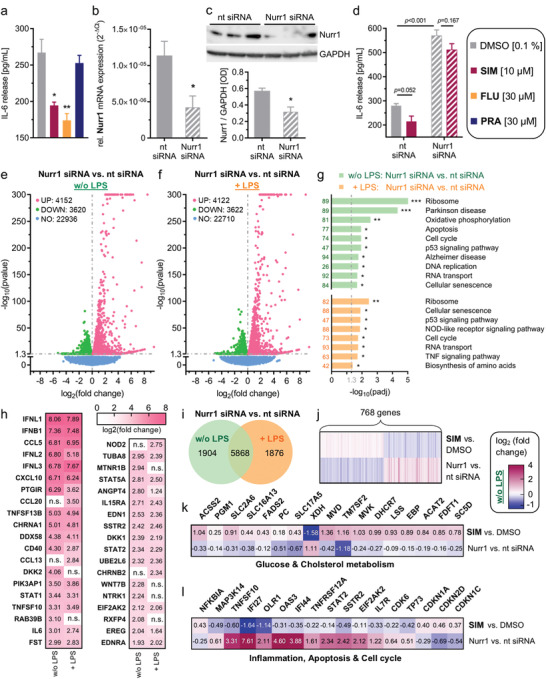
Nurr1 is involved in neuroinflammatory signaling and Nurr1 silencing causes opposite effects to simvastatin treatment. a) Lipopolysaccharide (LPS)‐treated (1 µg mL^−1^) T98G cells released considerable amounts of interleukin‐6 (IL‐6). The Nurr1 agonists simvastatin (SIM) and fluvastatin (FLU) significantly counteracted IL‐6 release from LPS‐treated T98G cells, whereas pravastatin (PRA), as negative control, did not affect IL‐6 release. Data are the mean ± S.E.M., *n* = 4, ^#^ *p* < 0.1, * *p* < 0.05, ** *p* < 0.01 (t‐test). b,c) Nurr1 knockdown efficiency as determined by Nurr1 mRNA levels (qRT‐PCR, 2^−ΔCt^ method with GAPDH as reference gene, b) and by western blot (c). Data are the mean ± S.E.M., *n* = 8 (qRT‐PCR), *n* = 4 (western blot), * *p* < 0.05 versus nontargeting (nt) control siRNA (t‐test). d) IL‐6 release from LPS‐treated T98G cells was further enhanced by siRNA‐mediated Nurr1 knockdown suggesting reverse Nurr1 involvement in this inflammatory response. The Nurr1 agonist SIM ameliorated the inflammatory response of T98G cells in a Nurr1‐dependent manner. Data are the mean ± S.E.M., *n* = 3, *p*‐values from t‐test. e,f) Differential gene expression in T98G cells treated with nt or Nurr1 siRNA in absence (e) or presence (f) of LPS. Volcano plots show log_2_fold change in gene expression level (*x*‐axis) versus statistical significance level (‐log_10_(*p*‐value); *y*‐axis). g) KEGG pathway enrichment analysis illustrates involvement of Nurr1 in signaling pathways related to neurodegenerative diseases and neuroinflammation. Bar plot shows statistical significance level (‐log_10_(padj)) of regulated KEGG pathways, numbers refer to the count of differentially expressed genes related to the pathway. *n* = 3, * *p* < 0.05, ** *p* < 0.01, *** *p* < 0.001. h) Selected differentially expressed genes with log_2_fold change > |2| associated with neurodegenerative diseases (PD, AD, neurodegeneration) or neuroinflammatory signaling (TNF, NFκB, WNT, TGFβ, JAK‐STAT, PI3K‐Akt, apoptosis, neuroactive interaction) according to KEGG are listed with their respective log_2_fold change values for Nurr1 silencing compared to nt siRNA control in absence or presence of LPS. n.s. – not significant. i) Coexpression Venn diagram for differential gene expression in Nurr1‐silenced cells versus nt siRNA for ± LPS‐treated cells. j–l) 768 genes were oppositely regulated by Nurr1 knockdown or simvastatin treatment in T98G cells (j, Gene list S3, Supporting Information) including several key genes of glucose and cholesterol metabolism (k) as well as inflammation, cell cycle regulation and apoptosis (l). Heatmaps show log_2_fold change for selected significantly (*p* < 0.05) regulated genes.

To probe a potential relevance of Nurr1 modulation by statins in neuroinflammation, we studied their effects on interleukin‐6 (IL‐6) release by Nurr1 expressing human astrocytes (T98G) in response to lipopolysaccharide (LPS) treatment. IL‐6 has pleiotropic functions with pro‐ and anti‐inflammatory roles depending on the site of action.^[^ ^28^ ^]^ Primary astrocytes and T98G cells have been shown to release IL‐6 upon inflammatory stimuli in vitro^[^ ^29^, ^30^ ^]^ suggesting IL‐6 as preliminary marker to study potential anti‐inflammatory effects of simvastatin and fluvastatin. Pravastatin, which does not activate Nurr1, was used as negative control. Simvastatin and fluvastatin markedly diminished LPS‐induced IL‐6 release while pravastatin had no effect suggesting Nurr1 involvement (**Figure** **3**a). Silencing of Nurr1 by RNAi in T98G cells (Figure 3b,c) remarkably increased IL‐6 production and abrogated the effect of simvastatin on IL‐6 levels (Figure 3d) further supporting Nurr1‐mediated activity of the statins.

### 2.4 Nurr1 Knockdown Alters Neuroinflammatory Signaling In Vitro

The pronounced effect of Nurr1 knockdown on IL‐6 levels aligned with the transcription factor's important role in neuroprotection and ‐inflammation. To obtain insights in its neuroprotective mechanisms in astrocytes, we studied differential gene expression of T98G cells treated with Nurr1 siRNA or nontargeting (nt) siRNA in presence or absence of LPS (Figure 3e–i). siRNA‐mediated Nurr1 knockdown altered the expression of almost 8000 genes in both untreated and LPS‐treated T98G cells but with pronounced differences of almost 2000 genes differentially affected by Nurr1 knockdown in presence or absence of LPS (Figure 3e,f,i; Gene list S1 and S2, Supporting Information). Pathway analysis of altered gene expression levels revealed strong effects of Nurr1 silencing on genes involved in PD and AD, oxidative phosphorylation, apoptosis, and p53 signaling (Figure 3g). Silencing of Nurr1 strongly increased expression of multiple cytokines (interferons, C–C and C–X–C chemokines), cytokine receptors, TNF superfamily genes (e.g., CD40), and members of JAK‐STAT signaling (STAT1, STAT2) even in absence of LPS stimulation (Figure 3h) indicating that diminished Nurr1 activity is sufficient to promote neuroinflammatory processes. Still, LPS treatment caused upregulation of additional cytokines (CCL13, CCL20) in Nurr1‐silenced cells.

### 2.5 Nurr1 Knockdown and Simvastatin Induce Opposite Gene Expression Changes

To evaluate potential Nurr1‐mediated neuroprotective effects of simvastatin, we compared the effect of simvastatin treatment and Nurr1 knockdown on gene expression. 768 genes were oppositely regulated upon simvastatin treatment or Nurr1 knockdown. 405 genes that were downregulated by Nurr1 knockdown were induced by simvastatin and 363 genes induced by Nurr1 knockdown were downregulated by simvastatin (Figure 3j; Gene list S3, Supporting Information). Additional LPS treatment increased the number of oppositely regulated genes to 872 (426 induced by simvastatin and downregulated by knockdown; 446 induced upon Nurr1 knockdown and suppressed by simvastatin; Gene list S4, Supporting Information). Pathway analysis revealed the most pronounced effects on gene expression of metabolic pathways. Interestingly, Nurr1 knockdown downregulated multiple genes involved in cholesterol biosynthesis (Figure 3k; e.g., MVD, TM7SF2, MVK, DHCR7, LSS) while these genes were induced by simvastatin including upstream enzymes of HMG‐CoA reductase (acetyl‐CoA acetyltransferase 2, ACAT2). Moreover, simvastatin and Nurr1 knockdown oppositely altered expression of genes involved in glucose utilization (PGM1, SLC2A6) and energy generation (ACSS2). Intriguingly, in addition to these beneficial metabolic effects, simvastatin caused anti‐inflammatory and anti‐apoptotic gene expression changes (Figure 3l) such as NFκB inhibitor α (NFKBIA) induction and downregulation of NFκB‐inducing kinase (MAP3K14), protein kinase R (EIF2AK2), tumor necrosis factor related apoptosis inducing ligand (TRAIL, TNFSF10), and interferon alpha‐inducible protein 27 (IFI27). Nurr1 knockdown exhibited the opposite activity suggesting cytoprotective effects of Nurr1 activation by simvastatin.

### 2.6 Simvastatin Induces Neuroprotective Gene Expression Changes via Nurr1

**Figure 4:**
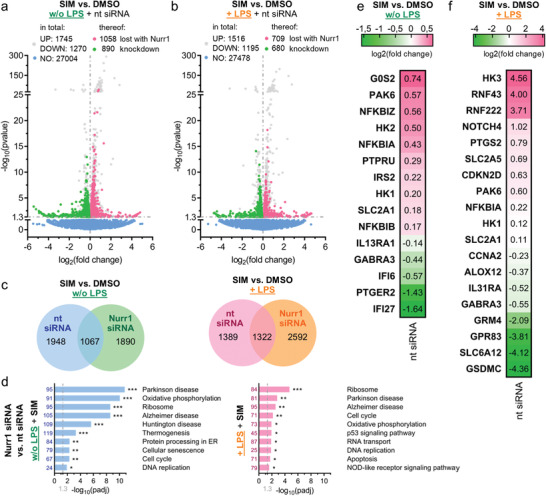
Simvastatin affected gene expression of human astrocytes (T98G) in a Nurr1‐dependent manner. a,b) Differentially expressed genes for simvastatin (SIM, 10 × 10^−6^ m) versus DMSO treatment in nt siRNA‐treated or Nurr1‐silenced T98G cells without (a) and with (b) additional lipopolysaccharide (LPS)‐stimulation. Genes regulated in nt siRNA‐treated and Nurr1‐silenced cells are gray, genes regulated only in nt siRNA‐treated but not in Nurr1‐silenced cells are red (induction) or green (downregulation). Volcano plots show log_2_fold change in gene expression level (*x*‐axis) versus statistical significance level (‐log_10_(*p*‐value); *y*‐axis), *n* = 3. c) Coexpression Venn diagrams show effects of SIM versus DMSO treatment in nt siRNA‐treated versus Nurr1‐silenced T98G cells with or without additional LPS‐stimulation. d) KEGG pathway enrichment analysis demonstrates effects of Nurr1 activation by SIM on the expression of genes related to cell cycle, apoptosis, oxidative phosphorylation, and thermogenesis as well as the neurodegenerative diseases AD and PD. Bar plot shows statistical significance level (‐log_10_(padj)) of regulated KEGG pathways, numbers refer to the count of differentially expressed genes related to the pathway. *n* = 3, * *p* < 0.05, ** *p* < 0.01, *** *p* < 0.001. e,f) Selected genes that were regulated by SIM versus DMSO treatment without (e) or with (f) additional LPS‐stimulation in nt siRNA‐treated cells whose expression was unaffected in Nurr1 siRNA‐treated cells. Only selected genes related to neuroprotection and neuroinflammation are shown, further regulated genes in Tables S1 and S2 of the Supporting Information. Heatmap shows log_2_fold change in gene expression.

The various opposite gene expression changes upon simvastatin treatment or Nurr1 knockdown (Figure 3j–l) pointed to several potential neuroprotective effects of Nurr1 modulation by simvastatin. To further elucidate these effects, we treated astrocytes (T98G) with nt siRNA or Nurr1 siRNA, with DMSO or simvastatin with or without LPS and determined differential gene expression (**Figure** **4**). Simvastatin caused multiple gene expression changes in all groups which revealed considerable differences between the nt siRNA and Nurr1 knockdown groups (Figure 4a–c). Effects of simvastatin on the expression of 1948 (without LPS) and 1389 (with LPS) genes in nt siRNA‐treated cells were lost in Nurr1‐silenced cells while 1067 (without LPS) and 1322 (with LPS) genes were regulated in both groups suggesting Nurr1‐dependent and independent mechanisms of simvastatin. Pathway analysis (Figure 4d) of nt siRNA versus Nurr1 siRNA‐treated cells stimulated with simvastatin demonstrated Nurr1‐mediated effects of simvastatin on genes related to cell cycle, apoptosis, oxidative phosphorylation and thermogenesis aligning with the observations from the comparison of simvastatin and Nurr1 knockdown effects on gene expression (compare Figure 3k,l). In addition, pronounced Nurr1‐mediated effects of simvastatin on genes related to AD and PD were evident from pathway analysis in both LPS‐treated and untreated cells.

Closer inspection of putatively Nurr1‐mediated effects of simvastatin revealed gene expression changes aligning with the hypothesized effects on inflammation, glucose and energy metabolism, and apoptosis and cell cycle regulation (Figure 4e,f; Tables S1 and S2, and Gene list S5 and S6, Supporting Information). In the context of glucose metabolism, simvastatin induced the expression of glucose transporters (SLC2A1, SLC2A5), insulin receptor substrate 2 (IRS2), and hexokinases (HK1, HK2, HK3) with the strongest effect observed for HK3 in LPS‐treated cells. HKs are rate‐limiting enzymes of glucose utilization and glucose is the main source of energy in the brain.^[^ ^31^ ^]^ Moreover, HKs have been associated with cytoprotective effects against oxidative stress, increased ATP levels and enhanced mitochondrial biogenesis.^[^ ^32^ ^]^ Glucose transporter and HK induction by simvastatin may hence importantly contribute to neuroprotective effects.

Nurr1‐mediated effects of simvastatin on genes involved in inflammation included induction of NFκB inhibitors alpha (NFKBIA), beta (NFKBIB), and zeta (NFKBIZ), and downregulation of arachidonate 12 lipoxygenase (ALOX12), IL‐13 receptor (IL13RA), IL‐31 receptor (IL31RA), and prostaglandin EP2 receptor (PTGER2). Interestingly, several anti‐inflammatory effects on gene expression were also evident in non‐LPS‐treated cells supporting a potential protective role of Nurr1 activation. In LPS‐treated cells, simvastatin additionally caused a remarkable suppression of gasdermin C (GSDMC) which is a membrane pore‐forming protein and a key mediator of inflammation and cell death.^[^ ^33^, ^34^, ^35^ ^]^ Gasdermin pores permeabilize cell membranes and damage mitochondria to release cytochrome C leading to inflammasome activation and enhanced apoptosis.^[^ ^33^, ^34^, ^35^ ^]^ Cyclooxygenase 2 (COX‐2, PTGS2) expression, by contrast, was not suppressed in LPS‐treated cells suggesting that Nurr1 activation did not fully block the LPS effects.

The differential gene expression data further revealed Nurr1‐mediated effects on cell cycle regulators with induction of CDKN2D, the G0/G1 switch 2 (G0S2), and the p21 activated kinase 6 (PAK6), and suppression of cyclin A2 (CCNA2). Additionally, simvastatin induced the receptor‐type tyrosine‐protein phosphatase PCP‐2 (PTPRU), which is known to be Nurr1 regulated,^[^ ^36^ ^]^ the E3‐ubiquitin ligases RING finger protein RNF43 and RNF222, and notch4. RNF43 is considered as an anti‐apoptotic regulator,^[^ ^37^ ^]^ as a Wnt antagonist^[^ ^38^ ^]^ and to be involved in DNA repair^[^ ^39^ ^]^ suggesting its upregulation as another neuroprotective contribution. Moreover, despite incomplete understanding of notch in neurodegeneration,^[^ ^40^ ^]^ decreased notch signaling has been detected in AD^[^ ^41^, ^42^ ^]^ indicating a potential benefit of notch induction by simvastatin.

Nurr1‐mediated effects of simvastatin on neurotransmitter receptors and transporters were also evident from the differential gene expression experiment. Simvastatin downregulated metabotropic glutamate receptor 4 (GRM4), GABA receptor A3 (GABRA3), the neuropeptide PEN receptor GPR83, the GABA transporter SLC6A12, and several ion channels (KCNA7, KCNB1, TRPV2).

Overall, differential gene expression and pathway analysis of simvastatin‐treated cells compared to simvastatin‐treated, Nurr1‐silenced cells further supported Nurr1‐mediated neuroprotective effects with anti‐inflammatory, metabolic, anti‐apoptotic, and cell cycle regulating contributions.

### 2.7 Promoter‐Specific Reporters Confirm Nurr1‐Mediated Effects of Simvastatin on Gene Expression

**Figure 5:**
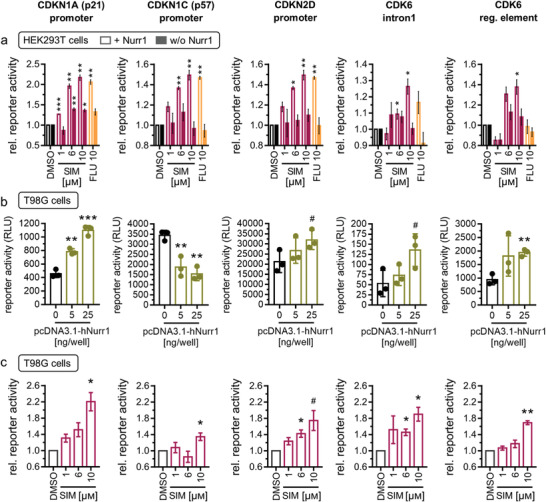
Reporter constructs validated Nurr1 involvement and Nurr1‐mediated effects of simvastatin in the regulation of CDKN1A (p21), CDKN1C (p57), CDKN2D, and CDK6. a) In presence of Nurr1 (plain bars), simvastatin (SIM, 1 × 10^−6^, 6 × 10^−6^, 10 × 10^−6^ m) and fluvastatin (FLU, 10 × 10^−6^ m) activated reporter constructs comprising the promoter regions of the CDKN1A, CDKN1C, and CDKN2D genes or the intron 1 of the CDK6 gene or the regulatory element of the CDK6 gene. Without cotransfection of Nurr1 (filled bars), the statins had a weaker or no effect. Data are the mean ± S.E.M., *n* = 4. b) In Nurr1 expressing T98G cells, Nurr1 overexpression altered reporter activity. Data are the mean ± SD, *n* = 3. c) Simvastatin activated the reporter constructs also in T98G cells. Data are the mean ± S.E.M., *n* = 4. ^#^ *p* < 0.1, * *p* < 0.05, ** *p* < 0.01, *** *p* < 0.001 (t‐tests vs control or as indicated).

From the differential gene expression analysis (Figures 3j–l and 4), regulation of genes involved in metabolism, inflammation, and cell cycle/apoptosis control was evident as potential Nurr1‐mediated neuroprotective mechanisms of simvastatin. Among 34 genes emerging with markedly different regulation by simvastatin treatment or Nurr1 knockdown (Figure 3k,l), sequence analysis revealed at least one Nurr1 response element (NBRE) within 2000 bp upstream from transcription start and the end of the 3′ untranslated region (UTR) in 17 genes (see the Experimental Section for details) supporting potentially direct involvement of Nurr1 in the regulation of these genes. To validate direct Nurr1‐mediated effects of simvastatin on the expression of differentially regulated critical genes in an orthogonal setting, we employed reporter constructs comprising the promoter regions of the CDKN1A (also known as p21), CDKN1C (also known as p57), and CDKN2D genes, the regulatory element of the CDK6 gene, or intron 1 of the CDK6 gene in front of a firefly luciferase gene to control reporter expression. In HEK293T cells, simvastatin activated all five reporters in a dose‐dependent fashion if Nurr1 was cotransfected (**Figure** **5**a, plain bars), and had a weaker or no effect in absence of Nurr1 (Figure 5a, filled bars). Fluvastatin revealed a similar profile.

In Nurr1 expressing T98G cells transfected with the reporter constructs, an involvement of Nurr1 in the regulation of CDKN1A and CDKN2D expression was evident from increased reporter activity upon Nurr1 overexpression (Figure 5b). Nurr1 overexpression also enhanced activity of the reporters for intron 1 and the regulatory element of the CDK6 gene which has a highly complex gene structure spanning 231 653 bp with only a 981 bp coding sequence suggesting complex regulation. Activation of the intron 1 and regulatory element driven reporters by Nurr1 overexpression supports relevance of the Nurr1 response elements in the CDK6 gene and the hypothesis that CDK6 is elongation‐regulated by Nurr1. Whether and how Nurr1 blocks or enhances elongation via such response elements after transcription remains to be elucidated, however. The activity of the CDKN1C reporter, despite being activated by Nurr1 in HEK293T cells, was diminished with increasing amounts of Nurr1 in T98G cells suggesting that additional factors to Nurr1 dominate its regulation in this cell type.

In line with the effects in HEK293T cells, simvastatin activated the CDKN1A, CDKN1C, CDKN2D, and CDK6 reporters in a dose‐dependent fashion also in T98G cells (Figure 5c). These findings confirm a direct involvement of Nurr1 in the regulation of CDKN1A, CDKN2D, and CDK6 as representative genes as well as Nurr1‐mediated effects of simvastatin on their expression. Despite validating only a subset of genes affected by simvastatin as direct Nurr1 targets, these results demonstrate that the effects of simvastatin on gene expression were at least in part directly mediated by Nurr1 activity and Nurr1 modulation by the drug.

### 2.8 Simvastatin‐Induced Gene Expression Changes Correlate with Biological Effects In Vitro

**Figure 6:**
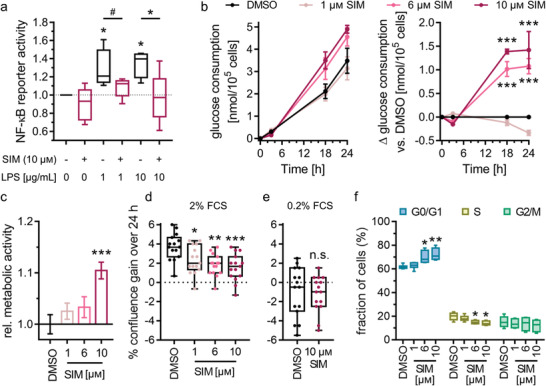
Simvastatin treatment altered inflammatory response, metabolism, and cell cycle of astrocytes. a) Simvastatin (SIM, 10 × 10^−6^ m) counteracted lipopolysaccharide (LPS)‐induced NFκB activity in T98G cells overexpressing Nurr1. Boxplots show min.–max. relative activity of an NFκB response element luciferase reporter, *n* = 4. ^#^ *p* < 0.1, * *p* < 0.05 (ANOVA). b) SIM enhanced glucose consumption of T98G cells in a dose‐dependent fashion. Graphs show mean ± S.E.M. absolute glucose consumption over time and mean ± S.E.M. Δglucose consumption versus DMSO‐treated cells over time, *n* = 3. *** *p* < 0.001 (ANOVA). c) SIM enhanced metabolic activity of T98G cells in a dose‐dependent fashion. Metabolic activity was determined with the WST‐8 reagent. Data are the mean ± S.E.M. relative metabolic activity versus DMSO‐treated cells, *n* = 15. *** *p* < 0.001 versus DMSO (ANOVA). d) SIM decreased the fetal calf serum (FCS)‐stimulated (2%) proliferation of T98G cells in a dose‐dependent fashion but had no effect in nonproliferating cells (0.2% FCS, e). Boxplots show min.–max. gain in confluence over 24 h, *n* = 15. * *p* < 0.05, ** *p* < 0.01, *** *p* < 0.001 versus DMSO‐treated cells (ANOVA). f) Cell cycle analysis of T98G cells treated with 2% FCS and varying concentrations of SIM by flow cytometry. Boxplots show min.–max., *n* = 6. * *p* < 0.05, ** *p* < 0.01 versus DMSO (ANOVA).

As the differential gene expression analysis revealed Nurr1‐mediated effects of simvastatin on genes involved in metabolism, proliferation, and inflammation as potential neuroprotective contributions we probed biological consequences of these gene expression changes. Anti‐inflammatory effects were evident from induction of NFKBIA expression and downregulation of MAP3K14, TNFSF10, IFI27, and IFI44 by simvastatin while Nurr1 knockdown caused the opposite effects (Figure 3k,l). Accordingly, simvastatin diminished IL‐6 release from LPS‐stimulated T98G cells (Figure 3a,d). This anti‐inflammatory activity was further confirmed by reduced NFκB activity upon simvastatin treatment (**Figure** **6**a). NFκB activity was stimulated by LPS (1, 10 µg mL^−1^) treatment in T98G cells and this effect was fully blocked by simvastatin (10 × 10^−6^ m) treatment which lowered NFκB activity to the level of non‐LPS‐treated T98G cells. Without LPS stimulation, simvastatin had no effect on NFκB activity. Together with multiple anti‐inflammatory gene expression changes induced by simvastatin and reversed by Nurr1 knockdown, reduced IL‐6 release and diminished NFκB activity in simvastatin‐treated astrocytes hence demonstrated anti‐inflammatory effects as neuroprotective mechanism of simvastatin with Nurr1 involvement.

In accordance with increased expression of genes involved in glucose utilization (PGM1, SLC2A6, HK3) and energy generation (ACSS2) upon simvastatin treatment, simvastatin also altered glucose consumption of T98G cells in a dose‐dependent fashion (Figure 6b). Cells treated with 6 or 10 × 10^−6^ m simvastatin consumed significantly more glucose over 24 h than cells treated with 1 × 10^−6^ m simvastatin or DMSO alone. Of note, the difference in glucose consumption was not detectable after 3 h of treatment but pronounced after 18 and 24 h suggesting that genomic mechanisms rather than enzyme or transporter inhibition were involved. In addition to enhanced glucose consumption, simvastatin‐treated cells exhibited increased metabolic activity (Figure 6c). Treatment with 10 × 10^−6^ m simvastatin elevated metabolic activity by 10%. These effects of simvastatin hence aligned with the observed gene expression changes in glucose transporting and metabolizing proteins and other factors of energy generation. Energy balance is crucial to neuronal health and glucose is the main source of energy in the brain.^[^ ^31^ ^]^ Contributions of altered glucose utilization and other metabolic factors to neurodegenerative diseases are well‐documented, especially in the context of AD.^[^ ^43^, ^44^, ^45^, ^46^, ^47^ ^]^ Improved glucose utilization and energy generation therefore evolve as further Nurr1‐mediated neuroprotective mechanisms of simvastatin.

Effects on cell cycle regulation implied by altered expression of CDK inhibitors (CDKN1A, CDKN1C, CDKN2D) and CDK6 upon simvastatin treatment were also evident in vitro from diminished fetal calf serum (FCS)‐stimulated proliferation of T98G cells treated with simvastatin (Figure 6d). The effect of simvastatin was dose‐dependent and conditional on the presence of FCS to stimulate proliferation (Figure 6e). Reduced proliferation was, however, not due to toxicity as confluence was not diminished by simvastatin at low FCS concentration (Figure 6e) and metabolic activity was not compromised but even increased (Figure 6c). Cell cycle analysis (Figure 6f) aligned with upregulation of CDK inhibitors (CDKN1A, CDKN1C, CDKN2D) and reduced proliferation upon simvastatin treatment. The drug induced a dose‐dependent increase of cells in the G0/G1 phase while cells in the S phase were concomitantly reduced (Figure 6f). These results suggest that simvastatin counteracted proliferation‐inducing stimuli (FCS) to reduce proliferation. Such effects have also been described as neuroprotective and anti‐neuroinflammatory mechanisms.^[^ ^48^, ^49^, ^50^, ^51^ ^]^ Cell cycle inhibition can prevent microglial activation^[^ ^49^, ^50^, ^52^ ^]^ and reduce neuronal injury.^[^ ^50^, ^53^ ^]^ Cell cycle inhibition may thus contribute as another neuroprotective mechanism to the effects of simvastatin.

Overall, the biological consequences of simvastatin treatment in vitro demonstrate effects of the drug on inflammation, (glucose) metabolism and cell cycle regulation and support an involvement of direct Nurr1‐mediated effects. These observations further endorse several Nurr1‐mediated neuroprotective mechanisms of simvastatin aligning with the observed gene expression changes. In addition to the anti‐inflammatory activity, beneficial metabolic effects and cell cycle inhibition, gene expression changes in several other pathways potentially suggest further neuroprotective mechanisms of simvastatin with involvement of Nurr1.

## 3 Conclusion

Several lines of evidence point to an important role and great therapeutic potential of the ligand sensing transcription factor Nurr1 in AD, PD, and MS but pharmacological validation and exploitation of Nurr1 as therapeutic target is pending. As the available collection of confirmed Nurr1 modulators is scarce, we aimed to rapidly expand the knowledge on Nurr1 ligand scaffolds and to provide new Nurr1 modulators as tools to probe the receptor's roles and potential. We have therefore screened a drug fragment collection for Nurr1 modulation and discovered remarkable Nurr1 agonism of statins. Simvastatin activated the human Nurr1 on all its three human response elements with low micromolar to sub‐micromolar potency and mechanistic characterization revealed displacement of NCoR‐1, NCoR‐2, NRIP1, and NCoA6 from the Nurr1 LBD, and decreased Nurr1 homodimerization as key molecular contributions to statin‐dependent Nurr1 activation.

The unprecedented molecular activity of the widely used drug class of statins on Nurr1 intriguingly aligns with several reports on neuroprotective effects of statins^[^ ^18^, ^19^, ^20^ ^]^ and suggests a potential involvement of Nurr1. Indeed, simvastatin counteracted inflammation in Nurr1 expressing astrocytes while this effect was lost in cells silenced for Nurr1 supporting relevance of Nurr1 activation by statins in neuronal cells. To capture effects of Nurr1 modulation by statins, we studied differential gene expression in Nurr1 expressing and silenced astrocytes upon simvastatin treatment. These experiments interestingly revealed strong proinflammatory effects of Nurr1 knockdown with markedly increased expression of multiple cytokines. Even more intriguing, the gene expression analysis suggested beneficial Nurr1‐mediated effects of simvastatin on inflammation, glucose utilization, energy generation, cell cycle regulation, and apoptosis. These effects of simvastatin treatment in astrocytes were diminished or lost in simvastatin‐treated cells upon Nurr1 knockdown and reverted in Nurr1‐silenced but not simvastatin‐treated cells although the Nurr1 knockdown was not complete. These observations support the hypothesis that simvastatin exhibits several neuroprotective mechanisms through Nurr1 modulation. Prominent gene expression changes evoked by simvastatin treatment, for example, induction of the cell cycle regulator p21 could be validated as directly Nurr1‐mediated with the help of reporter assays for the respective promoter regions. Moreover, we found that altered expression of genes involved in inflammation, metabolism, and cell cycle regulation translated into biological effects in simvastatin‐treated astrocytes, which displayed reduced LPS‐induced NFκB activity, enhanced glucose consumption and metabolic activity, and cell cycle inhibition with concomitantly diminished proliferation. Our results therefore demonstrate anti‐inflammatory activity, improved glucose and energy metabolism, and reduced proliferation as Nurr1‐mediated effects of simvastatin in vitro all of which can be regarded as neuroprotective mechanisms.^[^ ^31^, ^46^, ^47^, ^48^, ^49^, ^50^, ^51^, ^52^, ^53^ ^]^ Of note, simvastatin (and other statins) crosses the blood‐brain‐barrier^[^ ^54^ ^]^ suggesting a potential clinical relevance of Nurr1 activation in the CNS by statins. Despite the limitation that our results from an astrocyte‐like cell line lack confirmation in primary neurons and in vivo, our findings support the hypothesis that Nurr1 activation—together with other confirmed mechanisms^[^ ^55^, ^56^, ^57^, ^58^, ^59^ ^]^—is involved in the neuroprotective effects of statins.

Protective and therapeutic effects of statin treatment in neurodegenerative diseases have been reported by several studies.^[^ ^18^, ^19^, ^20^ ^]^ Particularly the use of simvastatin has been correlated with a suppression of proinflammatory molecules and microglial activation, inhibition of oxidative stress and attenuation of alpha‐synuclein aggregation.^[^ ^19^ ^]^ Important clinical evidence for therapeutic potential of statins was described by Wahner et al. who found protective effects for all statins except pravastatin against PD,^[^ ^60^ ^]^ which is particularly notable since pravastatin as only statin also failed to activate Nurr1. Observations on promising therapeutic potential in neurodegenerative diseases have evoked interventional clinical trials to reveal efficacy of simvastatin treatment in AD, PD, and MS. While the PD‐STAT^[^ ^61^ ^]^ trial could not confirm that simvastatin slows PD progression,^[^ ^62^ ^]^ impressive results on efficacy of simvastatin in secondary progressive MS have been reported from the MS‐STAT phase 2 trial.^[^ ^20^, ^63^ ^]^ Daily simvastatin treatment over two years significantly reduced brain atrophy compared to placebo and improved frontal lobe function and physical quality‐of‐life. The study concluded that its results support phase 3 testing but also noted that the mode‐of‐action for the observed neuroprotective effects of simvastatin in MS remains to be established. While HMG‐CoA reductase inhibition and improved cholesterol balance undoubtedly contribute to neuroprotective statin actions, there are also cholesterol‐independent activities the biochemical mechanisms of which remained elusive. Here, according to our observations, Nurr1 activation by statins evolves as a potentially critical mechanistic aspect in neuroprotective statin effects.

## 4 Experimental Section

### Chemicals and Compounds

All compounds tested in this study were obtained from commercial vendors (Prestwick Chemical Libraries, Illkirich, France; TCI Chemicals Deutschland GmbH, Eschborn, Germany; Sigma‐Aldrich, St. Louis, MO, USA; Alfa Aesar, Ward Hill, MA, USA; abcr GmbH, Karlsruhe, Germany; Cayman Chemical, Ann Arbor, MI, USA; Fluorochem Ltd., Glossop, United Kingdom; Oxchem Corp., Wood Dale, IL, USA).

### Hybrid Reporter Gene Assays

Plasmids: The Gal4‐fusion receptor plasmids pFA‐CMV‐hNURR1‐LBD, pFA‐CMV‐hPPARα‐LBD, pFA‐CMV‐hPPARγ‐LBD, pFA‐CMV‐hPPARδ‐LBD, pFA‐CMV‐hRARα‐LBD, and pFA‐CMV‐hRXRα‐LBD coding for the hinge region and LBD of the canonical isoform of the respective human nuclear receptor (NR) have been reported previously.^[^ ^14^ ^]^ The Gal4‐VP16^[^ ^22^ ^]^ fusion protein expressed from plasmid pECE‐SV40‐Gal4‐VP16^[^ ^21^ ^]^ (Addgene plasmid #71728, Watertown, MA, USA) served as ligand‐independent transcriptional inducer for control experiments. pFR‐Luc (Stratagene, La Jolla, CA, USA) was used as reporter plasmid and pRL‐SV40 (Promega, Madison, WI, USA) for normalization of transfection efficiency and test compound toxicity.

Assay procedure: HEK293T cells (ATCC® CRL‐3216™) were grown in DMEM high glucose, supplemented with 10% FCS, sodium pyruvate (1 × 10^−3^ m), penicillin (100 U mL^−1^), and streptomycin (100 µg mL^−1^) at 37 °C and 5% CO_2_. The day before transfection, HEK293T cells were seeded in 96‐well plates (3 × 10^4^ cells per well). Medium was changed to Opti‐MEM without supplements right before transfection. Transient transfection was performed using Lipofectamine LTX reagent (Invitrogen, Carlsbad, CA, USA) according to the manufacturer's protocol with pFR‐Luc (Stratagene), pRL‐SV40 (Promega), and the corresponding Gal4‐fusion nuclear receptor plasmid pFA‐CMV‐hNR‐LBD. 5 h after transfection, medium was changed to Opti‐MEM supplemented with penicillin (100 U mL^−1^) and streptomycin (100 µg mL^−1^), now additionally containing 0.1% DMSO and the respective test compound or 0.1% DMSO alone as untreated control. For the primary screen, each concentration was tested as single point measurements and each experiment was performed independently two times with 0.4% DMSO, respectively. For full dose‐response characterization, each concentration was tested in duplicates and each experiment was performed independently at least three times. The Gal4‐VP16 control experiment was carried out in duplicates as well, with at least four independent repeats. Following overnight (12–14 h) incubation with the test compounds, cells were assayed for luciferase activity using Dual‐Glo Luciferase Assay System (Promega) according to the manufacturer's protocol. Luminescence was measured with a Spark 10M luminometer (Tecan Group AG, Männedorf, Switzerland). Normalization of transfection efficiency and cell growth was done by division of firefly luciferase data by renilla luciferase data and multiplying the value by 1000 resulting in relative light units (RLU). Fold activation was obtained by dividing the mean RLU of a test compound at a respective concentration by the mean RLU of untreated control. Max. relative activation refers to fold reporter activation of a test compound divided by the fold activation of the respective reference agonist (PPARα: GW7647; PPARγ: rosiglitazone; PPARδ: L165041; RXRα: bexarotene; RARα: tretinoin; all at a concentration of 1 × 10^−6^ m; Nurr1: amodiaquine (100 × 10^−6^ m)). All hybrid assays were validated with the above mentioned reference agonists which yielded EC_50_ values in agreement with the literature. For dose‐response curve fitting and calculation of EC_50_/IC_50_ values, the equations “[Agonist]/[Inhibitor] versus response (three parameters)” were performed with mean fold activations ± S.E.M. using GraphPad Prism (version 7.00, GraphPad Software, La Jolla, CA, USA).

### Reporter Gene Assays Involving Full‐Length Human Nurr1

Plasmids: The reporter plasmids pFR‐Luc‐NBRE,^[^ ^14^ ^]^ pFR‐Luc‐NurRE,^[^ ^14^ ^]^ and pFR‐Luc‐DR5^[^ ^14^ ^]^ each containing one copy of the respective human Nurr1 response element NBRE Nl3 (TGATATCGAAAACAAAAGGTCA), NurRE (from POMC; TGATATTTACCTCCAAATGCCA) or DR5 (TGATAGGTTCACCGAAAGGTCA), were described previously. The full length human nuclear receptor Nurr1 (pcDNA3.1‐hNurr1‐NE; Addgene plasmid #102363) and, for DR5, RXRα (pSG5‐hRXR) were overexpressed. pFL‐SV40 (Promega) was used for normalization of transfection efficacy and evaluation of compound toxicity.

Assay procedure: HEK293T cells (ATCC® CRL‐3216™) were grown in DMEM high glucose, supplemented with 10% FCS, sodium pyruvate (1 × 10^−3^ M), penicillin (100 U mL^−1^) and streptomycin (100 µg mL^−1^) at 37 °C and 5% CO_2_. The day before transfection, HEK293T cells were seeded in 96‐well plates (3 × 10^4^ cells per well). Medium was changed to Opti‐MEM without supplements right before transfection. Transient transfection was performed using Lipofectamine LTX reagent (Invitrogen) according to the manufacturer's protocol with pFR‐Luc‐NBRE,^[^ ^14^ ^]^ pFR‐Luc‐NurRE^[^ ^14^ ^]^ or pFR‐Luc‐DR5,^[^ ^14^ ^]^ pRL‐SV40 (Promega), the human full length receptor plasmid pcDNA3.1‐hNurr1‐NE, and, for DR5, also pSG5‐hRXR. 5 h after transfection, medium was changed to Opti‐MEM supplemented with penicillin (100 U mL^−1^) and streptomycin (100 µg mL^−1^), now additionally containing 0.1% DMSO and the respective test compound or 0.1% DMSO alone as untreated control. For full dose‐response characterization, each concentration was tested in duplicates and each experiment was performed independently at least three times. Following overnight (12–14 h) incubation with the test compounds, cells were assayed for luciferase activity using Dual‐Glo Luciferase Assay System (Promega) according to the manufacturer's protocol. Luminescence was measured with a Spark 10M luminometer (Tecan Group AG). Normalization of transfection efficiency and cell growth was done by division of firefly luciferase data by renilla luciferase data and multiplying the value by 1000 resulting in RLU. Fold activation was obtained by dividing the mean RLU of a test compound at a respective concentration by the mean RLU of untreated control. The full length Nurr1 reporter gene assays were validated with amodiaquine and chloroquine as reference agonists. For dose–response curve fitting and calculation of EC_50_ values, the equation “[Agonist] versus response (three parameters)” was performed with mean fold activations ± S.E.M. using GraphPad Prism (version 7.00, GraphPad Software).

### Reporter Gene Assays for Promoter Clones

Sequence analysis: Genes identified as being differentially regulated in response to simvastatin or siRNA‐mediated Nurr1 knockdown were investigated for the presence of NR4A response elements. The entire genomic regions including ≥3 kb up‐ and downstream of these genes were downloaded from NCBI (updated annotation release 109.20211119 of NCBI homo sapiens annotation release 109). The program Geneious (version 11.1.2) was then used to search for Ad5 (CTCCAGCCTTGACCTT), DR5 (GGTTCACCGAAAGGTCA), DR5‐n5 (GGTTCANNNNNAGGTCA), DR5_con_‐n5 (RGKTCANNNNNAGGTCA), NBRE (AAAGGTCA), NurRE_con_ (TGACCTTTACCTCCAAAGGTCA), NurRE_con_‐n6 (TGACCTTTNNNNNNAAAGGTCA), and NurRE_POMC_ (TGATATTTACCTCCAAATGCCA) with ambiguity code according to IUPAC. Only instances of NBRE, which is the shortest sequence in the set and therefore statistically favored, were identified. Considering the entire genomic section from 2000 bp upstream of the transcription start (upts) to the end of the 3′UTR, NBRE was present in 17 out of 34 genes. In the OAS3 and CDKN2D genes, a copy of NBRE is present <400 bp upstream from transcription start supporting relevance of these NBRE motifs within the promoter regions. To probe a potential direct role of Nurr1 in the regulation of CDKN2D, a reporter construct for the CDKN2D promoter was cloned in pGL3‐CDKN2D promoter. Additionally, ten analyzed genes contain at least one copy of NBRE in intron sequences, SSTR2 contains one copy of NBRE in the 3′UTR, and four genes contain copies in at least one intron and in their 3′UTR, respectively. Among the latter is CDK6, which has a particularly complex gene structure spanning more than 230 kb. CDK6 intron 1 contains six copies of NBRE of which the first two were separated by less than 1 kb suggesting potential relevance. To study regulation by Nurr1, this section was cloned as pGL3‐CDK6 intron 1. Another two NBREs were in close proximity to an assigned enhancer region in the CDK6 gene that was predicted to comprise a transcriptional cis‐regulatory region (NFE2L2 motif). This section was cloned as pGL3‐CDK6 regE.

Cloning: Reporter clones for p21 (pGL2‐p21 promoter‐Luc,^[^ ^64^ ^]^ Addgene plasmid #33021) and p57 (pGL3‐p57,^[^ ^65^ ^]^ Addgene plasmid #101760) were obtained from Addgene. The vector pGL3 was amplified from pGL3‐p57 promoter‐Luc (Addgene plasmid #101760) using Q5 high‐fidelity DNA polymerase (NEB #M0491) with the primers 5′‐AAGCTTGGCATTCCGGTACTG‐3′ (fwd) and 5′‐GGTACCTATCGATAGAGAAATGTTCTGGC‐3′ (rev) to obtain the linearized vector backbone with blunt ends at the KpnI and HindIII cleavage sites present in the original multiple cloning site of empty pGL3‐basic (promega #E1751). For the construction of pGL3‐CDKN2D promoter, a slightly larger genomic section was first amplified from human genomic DNA (promega #G3041) using primers 5′‐CGGCTTTTTGCCTTTCCGTTT‐3′ (fwd) and 5′‐CAGTACCGGAATGCCAAGCTTCCTCCCTCCTCCTCG‐3′ (rev) which enabled specificity of the PCR reaction. The product was purified (QIAquick PCR purification, qiagen #28104) and subsequently used as template in a second PCR with primers 5′‐ ATTTCTCTATCGATAGGTACCTCTACTAAAAACACAAATTAGCCG ‐3′ (fwd) and 5′‐CAGTACCGGAATGCCAAGCTTCCTCCCTCCTCCTCG‐3′ (rev). After size selective purification via preparative DNA electrophoresis on a 1.5% agarose gel stained with crystal violet (Visual Violet, VWR, #N733‐KIT) followed by gel extraction (GenElute, Sigma‐Aldrich), the section encompassing 1023 bp upts to 33 after transcription start (ats) of the CDKN2D gene was cloned into the linearized pGL3 backbone by gibson assembly (NEBuilder HiFi DNA Assembly Master Mix, NEB #E2621). For the construction of pGL3‐CDK6 intron 1, two consecutive PCR reactions were performed. Primers 5′‐GCGCGATTATGCTATCCCCTT‐3′ (fwd) and 5′‐TTAGCCTTTTGGTTATGTTGCCT‐3′ (rev) were used for specific amplification from genomic DNA. The purified product was then used as template in the second PCR with primers 5′‐ATTTCTCTATCGATAGGTACCACATGCCTACTGTGTGCGCTG‐3′ (fwd) and 5′‐CAGTACCGGAATGCCAAGCTTCATGGCTCGTGACTGAGAGTC‐3′ (rev), which added the overhangs for gibson assembly to the insert encompassing 1951 bp ats to 3168 bp ats of the CDK6 gene. pGL3‐CDK6 regE was cloned by the same strategy, with initial amplification of a larger genomic section using primers 5′‐CAGGCTCGTTCACAATGCTTA‐3′ (fwd) and 5′‐CAGTACCGGAATGCCAAGCTTAGAACATAACCCATTTTCAGCTGG‐3′ (rev). The product was utilized as template in the second PCR with primers 5′‐ATTTCTCTATCGATAGGTACCACCACAAGCCTTTTGACTCCAG‐3′ (fwd) and 5′‐CAGTACCGGAATGCCAAGCTTAGAACATAACCCATTTTCAGCTGG‐3′ (rev). This insert comprises 23 561–25 225 bp ats and was purified as described before for Gibson assembly to obtain pGL3‐CDK6 regE.

Assay procedures: HEK293T cells (ATCC® CRL‐3216™) and T98G cells (ATCC® CRL1690™) were grown in DMEM high glucose, supplemented with 10% FCS, sodium pyruvate (1 × 10^−3^ m), penicillin (100 U mL^−1^), and streptomycin (100 µg mL^−1^) at 37 °C and 5% CO_2_. HEK293T cells were seeded in 96‐well plates (3 × 10^4^ cells per well). After 24 h, medium was changed to Opti‐MEM without supplements and cells were transiently transfected with one reporter clone and pRL‐SV40 with or without pcDNA3.1‐hNurr1‐NE using Lipofectamine LTX reagent (Invitrogen) according to the manufacturer's protocol. T98G cells were seeded in 96‐well plates (1.5 × 10^4^ cells per well). After 24 h, medium was changed to DMEM high glucose, supplemented with 0.2% FCS, sodium pyruvate (1 × 10^−3^ m), penicillin (100 U mL^−1^), and streptomycin (100 µg mL^−1^). After another 24 h, medium was changed to Opti‐MEM without supplements and cells were transiently transfected with one reporter clone and pRL‐SV40 with or without varying amounts of pcDNA3.1‐hNurr1‐NE using Lipofectamine 3000 reagent (Invitrogen) according to the manufacturer's protocol. 5 h after transfection, medium was changed to Opti‐MEM supplemented with penicillin (100 U mL^−1^) and streptomycin (100 µg mL^−1^), now additionally containing 0.1% DMSO and the respective concentration of simvastatin or fluvastatin or 0.1% DMSO alone as untreated control. Each sample was tested in duplicates and each experiment was performed independently at least three times. Following incubation for 16 h (HEK293T cells) or 20 h (T98G cells), cells were assayed for luciferase activity using Dual‐Glo Luciferase Assay System (Promega) according to the manufacturer's protocol. Luminescence was measured with a Spark 10M luminometer (Tecan Group AG). Normalization of transfection efficiency and cell growth was done by division of firefly luciferase data by renilla luciferase data and multiplying the value by 1000 resulting in RLU. Relative reporter activities were obtained by dividing the mean RLU of a test sample by the mean RLU of the untreated control.

### NFκB Activity Assay

T98G cells (ATCC® CRL1690™) were grown in DMEM high glucose, supplemented with 10% FCS, sodium pyruvate (1 × 10^−3^ m), penicillin (100 U mL^−1^), and streptomycin (100 µg mL^−1^) at 37 °C and 5% CO_2_ and seeded in 96‐well plates (1.5 × 10^4^ cells per well). After 24 h, medium was changed to DMEM high glucose, supplemented with 0.2% FCS, sodium pyruvate (1 × 10^−3^ m), penicillin (100 U mL^−1^), and streptomycin (100 µg mL^−1^). After another 24 h, medium was changed to Opti‐MEM without supplements and cells were transiently transfected with 4×NFkB Luc (Addgene plasmid #111216), pRL‐SV40 and pcDNA3.1‐hNurr1‐NE using Lipofectamine 3000 reagent (Invitrogen) according to the manufacturer's protocol. 5 h after transfection, medium was changed to Opti‐MEM supplemented with penicillin (100 U mL^–1^) and streptomycin (100 µg mL^−1^), additionally containing lipopolysaccharide (LPS, 0, 1, 10 µg mL^−1^), 0.1% DMSO and simvastatin (10 × 10^−6^ m) or 0.1% DMSO alone as untreated control. Each sample was tested in duplicates and each experiment was performed independently four times. Following incubation for 20 h, cells were assayed for luciferase activity using Dual‐Glo Luciferase Assay System (Promega) according to the manufacturer's protocol. Luminescence was measured with a Spark 10M luminometer (Tecan Group AG). Normalization of transfection efficiency and cell growth was done by division of firefly luciferase data by renilla luciferase data and multiplying the value by 1000 resulting in RLU. Relative NFκB activity was obtained by dividing the mean RLU of a test sample by the mean RLU of the untreated control.

### Glucose Consumption Assay

T98G cells (ATCC® CRL1690™) were grown in DMEM high glucose, supplemented with 10% FCS, sodium pyruvate (1 × 10^−3^ m), penicillin (100 U mL^−1^), and streptomycin (100 µg mL^−1^) at 37 °C and 5% CO_2_ and seeded in 12‐well plates (6 × 10^4^ cells per well). After 24 h, medium was changed to phosphate buffered saline (PBS) containing 0.1% DMSO and simvastatin (1 × 10^−6^, 6 × 10^−6^, 10 × 10^−6^ m) or 0.1% DMSO alone and cells were incubated at 37 °C and 5% CO_2_ for 2 h. Medium was then changed to 850 µL of a 4:1 mixture of PBS and DMEM low glucose medium supplemented with 10% FCS, sodium pyruvate (1 × 10^−3^ m), penicillin (100 U mL^−1^), and streptomycin (100 µg mL^−1^) additionally containing 0.1% DMSO and simvastatin (1 × 10^−6^, 6 × 10^−6^, 10 × 10^−6^ m) or 0.1% DMSO alone. 50 µL of supernatant was then sampled at time points 0, 3, 18, and 24 h, centrifuged at 14 000 × *g* for 5 min, snap‐frozen in liquid nitrogen and stored at −80 °C until further use. Glucose content in the sampled supernatants was quantified using a fluorometric assay (Abcam glucose assay kit #ab65333, Abcam, Cambridge, United Kingdom), following the manufacturer's instructions.

### Metabolic Activity Assay

T98G cells (ATCC® CRL1690™) were grown in DMEM high glucose, supplemented with 10% FCS, sodium pyruvate (1 × 10^−3^ m), penicillin (100 U mL^−1^), and streptomycin (100 µg mL^−1^) at 37 °C and 5% CO_2_ and seeded in 96‐well plates (1 × 10^4^ cells per well). After 24 h, medium was changed to DMEM high glucose, supplemented with 0.2% FCS, sodium pyruvate (1 × 10^−3^ m), penicillin (100 U mL^−1^), and streptomycin (100 µg mL^−1^). After 72 h, medium was changed to DMEM high glucose, supplemented with 0.2% FCS, sodium pyruvate (1 × 10^−3^ m), penicillin (100 U mL^−1^), and streptomycin (100 µg mL^−1^) additionally containing 0.1% DMSO and simvastatin (1 × 10^−6^, 6 × 10^−6^, 10 × 10^−6^ m) or 0.1% DMSO alone. After 18 h, medium was changed to DMEM high glucose, supplemented with 2% FCS, sodium pyruvate (1 × 10^−3^ m), penicillin (100 U mL^−1^), and streptomycin (100 µg mL^−1^) additionally containing 0.1% DMSO and simvastatin (1 × 10^−6^, 6 × 10^−6^, 10 × 10^−6^ m) or 0.1% DMSO alone. Metabolic activity of the cells was measured after 24 h using Cell Counting Kit‐8 (CCK‐8, #HY‐K0301, MedChem Express, NJ, USA), following the manufacturer's instructions. Absorbance was measured at 590 nm 60 min after addition of CCK‐8 solution on a Tecan Spark Cyto (Tecan Group AG).

### Proliferation Assay

T98G cells (ATCC® CRL1690™) were grown in DMEM high glucose, supplemented with 10% FCS, sodium pyruvate (1 × 10^−3^ m), penicillin (100 U mL^−1^), and streptomycin (100 µg mL^−1^) at 37 °C and 5% CO_2_ and seeded in 96‐well plates (1 × 10^4^ cells per well). After 24 h, medium was changed to DMEM high glucose, supplemented with 0.2% FCS, sodium pyruvate (1 × 10^−3^ m), penicillin (100 U mL^−1^), and streptomycin (100 µg mL^−1^). After 72 h, medium was changed to DMEM high glucose, supplemented with 0.2% FCS, sodium pyruvate (1 × 10^−3^ m), penicillin (100 U mL^−1^), and streptomycin (100 µg mL^−1^) additionally containing 0.1% DMSO and simvastatin (1 × 10^−6^, 6 × 10^−6^, 10 × 10^−6^ m) or 0.1% DMSO alone. After 18 h, medium was changed to DMEM high glucose, supplemented with 0.2% or 2% FCS, sodium pyruvate (1 × 10^−3^ m), penicillin (100 U mL^−1^), and streptomycin (100 µg mL^−1^) additionally containing 0.1% DMSO and simvastatin (1 × 10^−6^, 6 × 10^−6^, 10 × 10^−6^ m) or 0.1% DMSO alone. Cell confluence was assessed before and 24 h after adding 0.2% or 2% FCS supplemented medium on a Tecan Spark Cyto (Tecan Group AG). Cell growth was expressed as change in confluency between time points 0 and 24 h.

### Cell Cycle Analysis

T98G cells (ATCC® CRL1690™) were grown in DMEM high glucose, supplemented with 10% FCS, sodium pyruvate (1 × 10^−3^ m), penicillin (100 U mL^−1^), and streptomycin (100 µg mL^−1^) at 37 °C and 5% CO_2_ and seeded in 12‐well plates (1 × 10^5^ cells per well). After 24 h, medium was changed to DMEM high glucose, supplemented with 0.2% FCS, sodium pyruvate (1 × 10^−3^ M), penicillin (100 U mL^−1^), and streptomycin (100 µg mL^−1^) to achieve cell cycle synchronization over 72 h. Medium was then changed to DMEM high glucose, supplemented with 0.2% FCS, sodium pyruvate (1 × 10^−3^ m), penicillin (100 U mL^−1^), and streptomycin (100 µg mL^−1^) additionally containing 0.1% DMSO and simvastatin (1 × 10^−6^, 6 × 10^−6^, 10 × 10^−6^ m) or 0.1% DMSO alone. After 18 h, medium was changed to DMEM high glucose, supplemented with 0.2% or 2% FCS, sodium pyruvate (1 × 10^−3^ m), penicillin (100 U mL^−1^), and streptomycin (100 µg mL^−1^) additionally containing 0.1% DMSO and simvastatin (1 × 10^−6^, 6 × 10^−6^, 10 × 10^−6^ m) or 0.1% DMSO alone. After 24 h, cells were harvested using trypsin/EDTA, washed once with PBS, and fixed by dropwise addition of 1 mL ice‐cold 70% ethanol. After incubation for 30 min on ice, cells were centrifuged at 900 × *g* for 5 min, washed once with cold PBS and again centrifuged. Cells were then resuspended in 200 µL FxCycle PI/RNase Staining Solution (#F10797, Invitrogen) and incubated for 30 min at room temperature. Propidium iodide signal intensity per cell was assessed using a BD Canto II flow cytometer (BD Biosciences, NJ, USA) and cell cycle analysis was performed using Floreada.io software.

### Nurr1 Coregulator Recruitment Assays

Recruitment of coregulator peptides to the Nurr1‐LBD was studied in a homogeneous time‐resolved fluorescence resonance energy transfer (HT‐FRET) assay system. Terbium cryptate as streptavidin conjugate (Tb‐SA; Cisbio Bioassays, Codolet, France) was used as FRET donor for stable coupling to biotinylated recombinant Nurr1‐LBD protein,^[^ ^14^ ^]^ which has been reported previously. Four coregulator peptides fused to fluorescein as FRET acceptor were purchased from ThermoFisher Scientific (Life Technologies GmbH, Darmstadt, Germany). Assay solutions were prepared in HTRF assay buffer (25 × 10^−3^ m HEPES pH 7.5, 150 × 10^−3^ m KF, 5% (w/v) glycerol, 5 × 10^−3^ m DTT) supplemented with 0.1% (w/v) CHAPS and contained recombinant biotinylated Nurr1‐LBD (3 × 10^−9^ m), Tb‐SA (3 × 10^−9^ m) and the respective fluorescein‐labeled coregulator peptide (100 × 10^−9^ m) as well as 1% DMSO with varying concentrations of the test compounds simvastatin or fluvastatin, or DMSO alone as negative control. All HTRF experiments were carried out in 384 well format using white flat bottom polystyrol microtiter plates (Greiner Bio‐One, Frickenhausen, Germany). All samples were set up in triplicates. After 2 h incubation at RT, fluorescence intensities (FIs) after excitation at 340 nm were recorded at 520 nm for fluorescein acceptor fluorescence and 620 nm for Tb‐SA donor fluorescence on a SPARK plate reader (Tecan Group AG). FI520nm was divided by FI620nm and multiplied with 10 000 to give a dimensionless HTRF signal. The coregulator peptides in this experiment were the following: nuclear receptor corepressor (NCoR) 1, fluorescein‐RTHRLITLADHICQIITQDFARN‐OH; NCoR‐2, fluorescein‐HASTNMGLEAIIRKALMGKYDQW‐OH; nuclear receptor coactivator 6 (NCoA6) fluorescein‐VTLTSPLLVNLLQSDISAG‐OH; nuclear receptor interacting protein 1 (NRIP1), fluorescein‐SHQKVTLLQLLLGHKNEEN‐OH. For dose‐response curve fitting and calculation of IC_50_ values, the equation “[Inhibitor] versus response–Variable slope (four parameters)” was performed with mean fold activations ± SD using GraphPad Prism (version 7.00, GraphPad Software).

### Nurr1 Dimerization Assays

Modulation of Nurr1 LBD homodimerization and heterodimerization with RXRα LBD were studied in HT‐FRET assay setups using biotinylated recombinant Nurr1‐LBD^[^ ^14^ ^]^ and GFP‐Nurr1 LBD^[^ ^14^ ^]^ or GFP‐RXRα LBD,^[^ ^14^ ^]^ respectively. Assay solutions were prepared in HTRF assay buffer supplemented with 0.1% (w/v) CHAPS as well as 1% DMSO with test compounds at 30 × 10^−6^ m or DMSO alone as negative control. The biotinylated Nurr1 LBD (0.375 × 10^−9^ m) and Tb‐SA (0.75 × 10^−9^ m) served as FRET donor complex which was kept constant while the GFP‐coupled protein as FRET acceptor was varied in concentration. Since affinity of both Nurr1 dimer formations differ, titration of GFP‐Nurr1 LBD started at 500 × 10^−9^ m and, for GFP‐RXRα LBD, at 4 × 10^−6^ m, respectively. Accordingly, free GFP was added to keep the total GFP content stable throughout the entire series in order to suppress artefacts from changes in degree of diffusion enhanced FRET. All samples were set up in triplicates and equilibrated at RT for 2 h before FI520 and FI620 were recorded after excitation at 340 nm, and the HTRF signal was calculated as described above.

### Nurr1 Knockdown in T98G Cells

T98G cells (ATCC® CRL1690™) were grown in DMEM high glucose, supplemented with 10% FCS, sodium pyruvate (1 × 10^−3^ m), penicillin (100 U mL^−1^), and streptomycin (100 µg mL^−1^) at 37 °C and 5% CO_2_. 24 h before transfection, T98G cells were seeded in 12‐well plates (1 × 10^5^ cells per well). The medium was changed to reduced serum medium containing DMEM high glucose supplemented with 1% charcoal‐stripped FCS and sodium pyruvate (1 × 10^−3^ m) right before transfection. Knockdown was mediated by transient transfection using the RNAiMAX reagent (Invitrogen) according to the manufacturer's protocol with 30 × 10^−9^ m of Nurr1 targeting esiRNA (Cat# EHU008731) or nontargeting control siRNA (Cat# SIC001, both Sigma‐Aldrich). 24 h after transfection, the cells were rather harvested and directly used for RNA extraction or used for further experiments. 2 µg of total RNA was extracted from T98G cells by the E.Z.N.A. Total RNA Kit I (R6834‐02, Omega Bio‐Tek, Inc., Norcross, GA). RNA was reverse‐transcribed into cDNA using the High‐Capacity RNA‐to‐cDNA Kit (4387406, Thermo Fischer Scientific Inc., Waltham, MA, USA) according to the manufacturer's protocol. Nurr1 knockdown efficiency was evaluated by quantitative real‐time PCR (qRT‐PCR) analysis with a StepOnePlus System (Life Technologies, Carlsbad, CA) using Power SYBR Green (Life Technologies; 12.5 µL per well). Each sample was set up in duplicates and repeated in eight independent experiments. The expression was quantified by the comparative 2^−ΔCt^ method and glyceraldehyde 3‐phosphate dehydrogenase (GAPDH) served as the reference gene. Primer sequences for the human Nurr1 gene were obtained from OriGene (OriGene Technologies Inc., Rockville, MD, USA). The following PCR primers were used: hGAPDH: 5′‐ATA TGA TTC CAC CCA TGG CA (fw), 5′‐GAT GAT GAC CCT TTT GGC TC (rev), hNurr1: 5′‐AAA CTG CCC AGT GGA CAA GCG T (fw), 5′‐GCT CTT CGG TTT CGA GGG CAA A (rev). Nurr1 knockdown efficiency was additionally validated by western blot. For this, cells were harvested 24 h after siRNA transfection using trypsin/EDTA, resuspended in 100 µL complete radioimmunoprecipitation assay buffer (10 mL Pierce RIPA buffer supplemented with 1 tablet Pierce Protease and Phosphatase Inhibitor, Thermo Fisher #A32959), and incubated at 4 °C and 600 rpm horizontal shaking for 15 min. After 10 min centrifugation at 14 000 × *g* and 4 °C, supernatant was harvested, mixed with 25 µL 5× Pierce TM Lane Marker Reducing Sample Buffer (Thermo Fisher # 39000), and boiled at 95 °C for 5 min. Samples were then stored at −80 °C until further processing. Sodium dodecyl sulfate (SDS) polyacrylamide gel electrophoresis was conducted using 4–12% gradient gels (#43273.01, SERVA Electrophoresis, Heidelberg, Germany) loaded with 25 µL protein extract at 100 V for 20 min and 200 V for 50 min in running buffer (25 × 10^−3^ m TRIS, 192 × 10^−3^ m glycine, 0.1% w/v SDS, pH 8.3). Right before semidry transfer of the separated protein to a methanol‐activated polyvinylidene fluoride (PVDF) membrane (Immobilon‐FL PVDF‐Membrane, #05317, Merck Millipore, MA, USA), gel and membrane were equilibrated in transfer buffer (12.5 × 10^−3^ m TRIS, 80 × 10^−3^ m glycine, 20% v/v methanol) for 20 min. Semidry transfer using transfer buffer drenched Whatman‐paper (Whatman plc, Maidstone, United Kingdom) was conducted at 10 V and 1.5 mA cm^−^^2^ for 1 h. The PVDF membrane was washed four times for 10 min in Tris‐buffered saline with 0.5% Tween 20 (TBST) and incubated in TBST with 5% skimmed milk and anti‐NR4A2 (diluted 1:3000, #N6413, Sigma‐Aldrich) or anti‐GAPDH (diluted 1:2000, clone D16H11, Cell Signaling Technology, MA, USA) over night at 4 °C, respectively. After repeating the washing step as described above, the membrane was incubated with horseradish peroxidase‐conjugated secondary antibody (Sigma‐Aldrich #12‐348, diluted 1:4000 in TBST and 5% skimmed milk) for 1 h at room temperature. After washing in TBST, the membrane was submerged in enhanced chemiluminescence solution (100 × 10^−3^ m Tris/HCl pH 8.8, 2.5 × 10^−3^ m luminol, 0.4 × 10^−3^ m p‐coumaric acid, 2.6 × 10^−3^ m hydrogen peroxide) for 1 min and signal was detected using a ChemiDoc Imaging System (BioRad Laboratories Inc., CA, USA).

### Quantification of IL‐6 Release in T98G Cells

T98G (ATCC® CRL1690™) cells were grown in 12‐well plates (1 × 10^5^ cells per well) for knockdown experiments or 24‐well plates (5 × 10^4^ cells per well) in DMEM high glucose, supplemented with 10% FCS, sodium pyruvate (1 × 10^−3^ m), penicillin (100 U mL^−1^), and streptomycin (100 µg mL^−1^) at 37 °C and 5% CO_2_ for 24 h. Before incubation with LPS and test compounds, the medium was changed to DMEM supplemented with 1% charcoal‐stripped FCS, sodium pyruvate (1 × 10^−3^ m), penicillin (100 U mL^−1^), and streptomycin (100 µg mL^−1^) for 12 h, or Nurr1 knockdown was performed by transient transfection for 24 h as outlined above. The cells were then treated with LPS (1 µg mL^−1^) to induce inflammation and simultaneously incubated with simvastatin (10 × 10^−6^ m), fluvastatin (30 × 10^−6^ m) or pravastatin (30 × 10^−6^ m), and 0.1% DMSO, or 0.1% DMSO alone as untreated control. Each sample was repeated independently at least three times. Following overnight (12 h for knockdown or 24 h) incubation, 100 µL of the respective supernatants was collected and assayed for IL‐6 using the Human IL‐6 ELISA Kit (Cat# KHC0061, Thermo Fisher Scientific, Inc.) according to the manufacturer's protocol. Absorbance at 450 nm was measured with a Spark 10M luminometer (Tecan Group AG).

### Differential Gene Expression Analysis

Sample preparation: T98G cells (ATCC® CRL1690™) were cultured in DMEM, high glucose supplemented with 10% FCS, sodium pyruvate (1 × 10^−3^ m), penicillin (100 U mL^−1^), and streptomycin (100 µg mL^−1^) at 37 °C and 5% CO_2_ and seeded in 12‐well plates (1 × 10^5^ cells per well) for gene expression analysis. 24 h after seeding, medium was changed to reduced serum medium containing DMEM high glucose supplemented with 1% charcoal‐stripped FCS, sodium pyruvate (1 × 10^−3^ m), penicillin (100 U mL^−1^), and streptomycin (100 µg mL^−1^) right before transfection. Knockdown was mediated by transient transfection using the RNAiMAX reagent (Invitrogen) according to the manufacturer's protocol with 30 × 10^−9^ m of Nurr1 targeting esiRNA (Cat# EHU008731) or nontargeting control siRNA (Cat# SIC001, both from Sigma‐Aldrich). After further 24 h, medium was changed again to reduced serum medium supplemented as described above now additionally containing 0.1% DMSO and the test compound simvastatin (10 × 10^−6^ m) or 0.1% DMSO alone as control. Additionally, LPS (1 µg mL^–1^) was added simultaneously to induce inflammation in one treatment arm. Each condition was set up in three independent biological repeats (*n* = 3). After 12 h incubation, cells were harvested, washed twice with cold PBS and then directly used for RNA extraction by the E.Z.N.A. Total RNA Kit I (R6834‐02, Omega Bio‐Tek Inc., Norcross, GA, USA).

mRNA sequencing: A total amount of 1 µg RNA per sample was used as input material for the RNA sample preparations. Sequencing libraries were generated using NEBNext Ultra RNA Library Prep Kit for Illumina (New England Biolabs (NEB), Ipswich, MA, USA) following manufacturer's recommendations and index codes were added to attribute sequences to each sample. Briefly, mRNA was purified from total RNA using poly‐T oligo‐attached magnetic beads. Fragmentation was carried out using divalent cations under elevated temperature in NEBNext First Strand Synthesis Reaction Buffer (5×). First strand cDNA was synthesized using random hexamer primer and M‐MuLV Reverse Transcriptase (RNase H‐). Second strand cDNA synthesis was subsequently performed using DNA Polymerase I and RNase H. Remaining overhangs were converted into blunt ends via exonuclease/polymerase activities. After adenylation of 3′ ends of DNA fragments, NEBNext Adaptor with hairpin loop structure was ligated to prepare for hybridization. In order to select cDNA fragments of preferentially 150–200 bp in length, the library fragments were purified with AMPure XP system (Beckman Coulter, Beverly, USA). Then 3 µL USER Enzyme (NEB, USA) was used with size‐selected, adaptor ligated cDNA at 37 °C for 15 min followed by 5 min at 95 °C before PCR. Then PCR was performed with Phusion High‐Fidelity DNA polymerase, Universal PCR primers and Index (X) Primer. At last, PCR products were purified (AMPure XP system) and library quality was assessed on the Agilent Bioanalyzer 2100 system. The clustering of the index‐coded samples was performed on a cBot Cluster Generation System using PE Cluster Kit cBot‐HS (Illumina) according to the manufacturer's instructions. After cluster generation, the library preparations were sequenced on an Illumina NovaSeq 6000 platform and paired‐end reads were generated.

Data analysis: Raw data (raw reads) of FASTQ format were first processed through fastp.^[^ ^66^ ^]^ In this step, clean data (clean reads) were obtained by removing reads containing adapter and poly‐N sequences and reads with low quality from raw data. At the same time, Q20, Q30, and GC content of the clean data were calculated. All the downstream analyses were based on the clean data with high quality. Downstream analysis was performed using a combination of programs including STAR,^[^ ^67^ ^]^ HTseq,^[^ ^68^ ^]^ Cufflink,^[^ ^69^ ^]^ and wrapped scripts. Alignments were parsed using TopHat program^[^ ^69^ ^]^ and differential expressions were determined through DESeq2.^[^ ^70^ ^]^ Kyoto Encyclopedia of Genes and Genomes (KEGG) enrichment analysis was implemented by the ClusterProfiler. Reference genome and gene model annotation files were downloaded from genome website browser (NCBI/UCSC/Ensembl) directly. Indexes of the reference genome were built using STAR and paired‐end clean reads were aligned to the reference genome using STAR (v2.5). STAR used the method of Maximal Mappable Prefix, which can generate a precise mapping result for junction reads. HTSeq v0.6.1 was used to count the read numbers mapped of each gene. Reads per kilobase of exon model per million mapped reads (RPKM, considering the effect of sequencing depth and gene length for the reads count at the same time) of each gene was calculated based on the length of the gene and reads count mapped to this gene. Differential expression analysis between two conditions/groups (three biological repeats per condition) was performed using the DESeq2 R package (2_1.6.3). DESeq2 provides statistical routines for determining differential expression in digital gene expression data using a model based on the negative binomial distribution. The resulting *p*‐values were adjusted using the Benjamini and Hochberg's approach for controlling the false discovery rate. Genes with an adjusted *p*‐value < 0.05 found by DESeq2 were assigned as differentially expressed. Venn diagrams were prepared using the function vennDiagram in R based on the gene list for different group. ClusterProfiler R package^[^ ^71^ ^]^ was used to test the statistical enrichment of differential expression of genes in KEGG pathways using the KEGG database resource (http://www.genome.jp/kegg/).^[^ ^72^ ^]^ Correlations between individual samples were determined using the cor.test function in R with options set alternative = “greater” and method = “Spearman”. To identify the correlation between differences, different samples were clustered by expression level RPKM using hierarchical clustering distance method with the function heatmap, self‐organization mapping and kmeans using silhouette coefficient to adapt the optimal classification with default parameter in R.

### Statistical Analysis

Experiments were conducted in at least three biologically independent repeats in technical duplicates. Exact sample sizes are given in the methods and in the figure captions (Figure 1, 2, 3, 4, 5, 6; Figure S1–S4). Data from cellular assays were normalized to DMSO‐treated cells (negative control) where specified. Data are presented as mean ± SD or mean ± S.E.M. as specified in the figure captions. Statistical analysis was performed in R and GraphPad Prism. Statistical significance was evaluated over biological repeats using the mean of technical duplicates. Data were checked for normal distribution (Shapiro‐Wilk test), homoscedasticity (Levene's test), and outliers (Grubb's test) prior to other statistical analysis. Normally distributed and homoscedastic data sets were tested for statistically significant differences via multiple t‐tests (two‐sided) or by ANOVA with post hoc Bonferroni's correction for multiple comparisons. *p*‐Values < 0.05 were considered as statistically significant, *p* < 0.1 was considered as a trend. Statistical analysis of the differential gene expression experiment is described in the Differential Gene Expression Analysis section.

## Conflict of Interest

The authors declare no conflict of interest.

## Supporting information

Supporting Information

Supplemental Gene list 1

Supplemental Gene list 2

Supplemental Gene list 3

Supplemental Gene list 4

Supplemental Gene list 5

Supplemental Gene list 6

## Data Availability

The data that support the findings of this study are openly available in ArrayExpress at https://www.ebi.ac.uk/arrayexpress/, reference number 10624.
