# Supplementary material for: Nurr1 Modulation Mediates Neuroprotective Effects of Statins
Source: Adv Sci (Weinh). 2022 Apr 30;9(18):2104640. doi: 10.1002/advs.202104640 (PMC9218776; doi:10.1002/advs.202104640)
Supplement: Supplementary file 1 — Supporting Information [file ADVS-9-2104640-s005.pdf]

Supporting Information

Nurr1 modulation mediates neuroprotective effects of statins

*Sabine Willems, Julian Marschner, Whitney Kilu, Giuseppe Faudone, Romy Busch, Silke Duensing-Kropp, Jan Heering, Daniel Merk**

S. Willems, W. Kilu, G. Faudone, Prof. Dr. D. Merk
Goethe University Frankfurt, Institute of Pharmaceutical Chemistry, Max-von-Laue-Str. 9, 60438 Frankfurt, Germany

J. Heering
Fraunhofer Institute for Translational Medicine and Pharmacology ITMP, Theodor-Stern-Kai 7, 60596 Frankfurt, Germany

Dr. J. Marschner, R. Busch, S. Duensing-Kropp, Prof. Dr. D. Merk
Ludwig-Maximilians-Universität München, Department of Pharmacy, Butenandtstr. 5-13, 81377 Munich, Germany
E-Mail: daniel.merk@cup.lmu.de

Table of contents

Supporting Figures and Tables.. 2

Supporting Methods 13

Supporting References 14

Supporting Figures and Tables

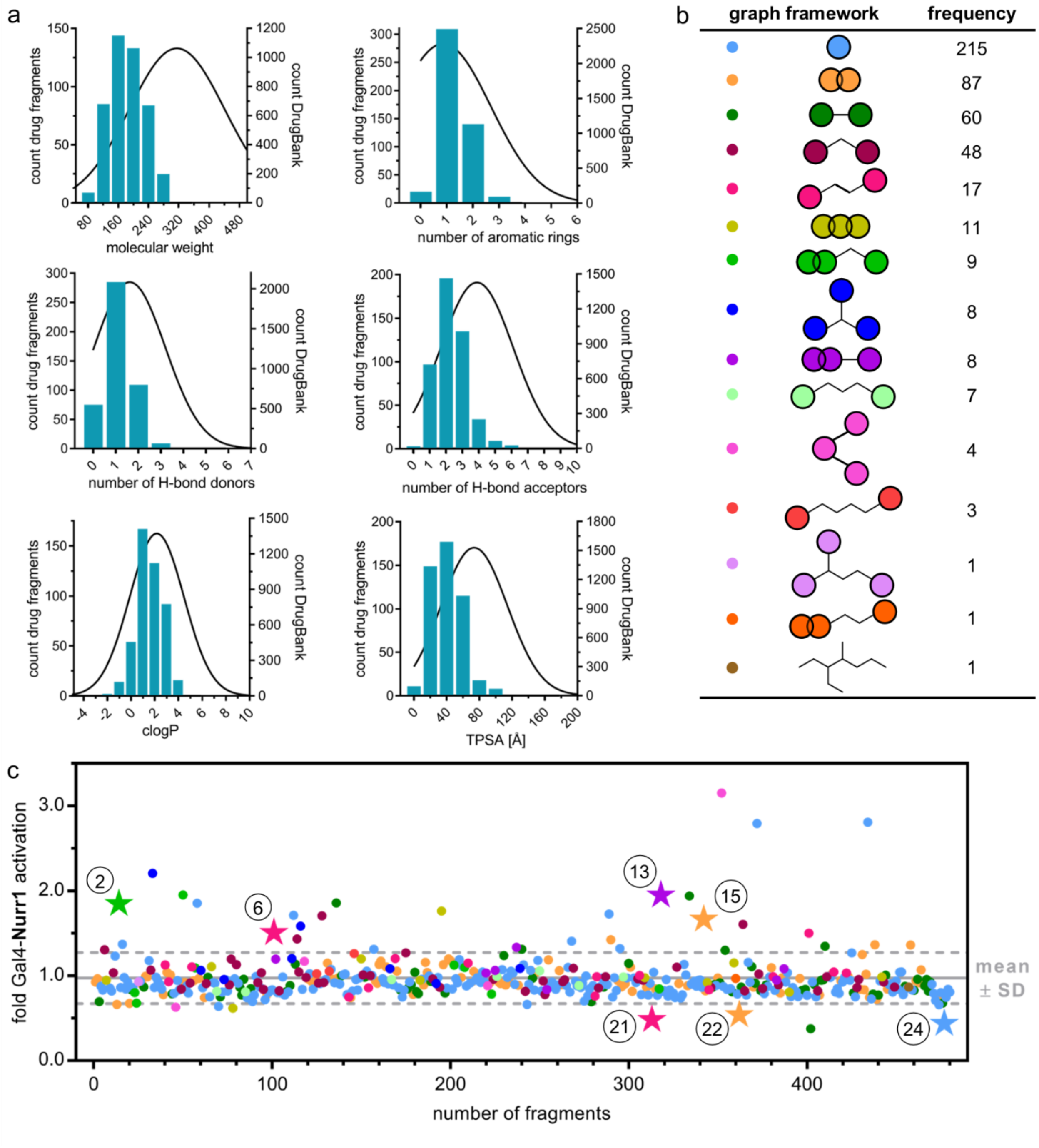

Figure S1. (A) Feature distributions of the drug fragment library (N=480) in comparison with the DrugBank database (N=7946). Bars are plotted on the left y-axis for Prestwick drug fragments. Gaussian distributions of DrugBank compounds (MW ≤ 500) are plotted on the right y-axis. (B) Representations of the different graph frameworks contained in the drug fragment library and their frequencies. (C) Nurr1 modulatory activity of the drug fragment library in a Gal4 hybrid Nurr1 reporter gene assay. Results from primary screen are the mean reporter activity vs. 0.4% DMSO; n=2. Fragments affecting reporter activity ≥1.5-fold (Nurr1 activation, **1-20**) or ≤0.6-fold (Nurr1

repression, **21**-**24**) were considered for further evaluation as primary screening hits. Labeled compounds marked with a star relate to the fragment hits validated in control experiments on Gal4-VP16. Different colors refer to different graph frameworks (from B). Gray lines represent mean±SD of the entire screening.

a

fragment hit	structure	primary screen (reporter act. at 100 µM)	Gal4-Nurr1 modulation
1	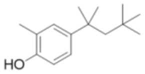	4.16±5.23	toxic in primary screen
2	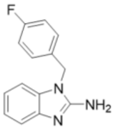	1.85±0.04	EC_{50} 11±6 (1.98±0.21 max. act.)
3	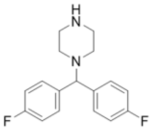	2.21±0.83	inactive (50µM) toxic (≥ 100 µM)
4	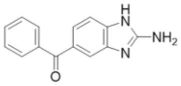	1.95±0.33	inactive (50 µM) toxic (≥ 100 µM)
5	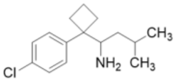	1.85±0.31	inactive (30 µM) toxic (≥ 50 µM)
6	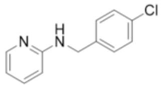	1.51±0.36	EC_{50} 39±14 (1.82±0.14 max. act.)
7	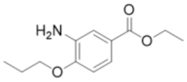	1.71±0.07	inactive (50 µM) toxic (≥ 100 µM)
8	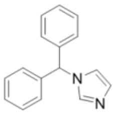	1.59±0.29	inactive (30 µM) toxic (≥ 50 µM)
9	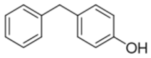	1.71±0.02	toxic in primary screen
10	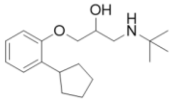	1.86±0.43	inactive (30 µM) toxic (≥ 100 µM)
11	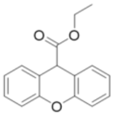	1.76±0.10	inactive(50 µM) toxic (≥ 100 µM)
12	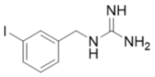	1.73±0.35	toxic in primary screen
13	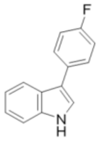	1.95±1.44	EC_{50} 7.7±3.2 (2.48±0.18 max. act.)
14	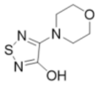	1.94±0.24	inactive (10 µM) toxic (≥ 30 µM)
15	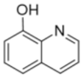	1.66±0.33	EC_{50} 25±10 (1.89±0.14 max. act.)
16	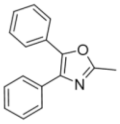	3.15±0.79	inactive (10 µM) toxic (≥ 30 µM)
17	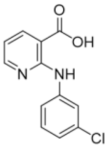	1.60±0.23	inactive(10 µM) toxic (≥ 30 µM)
18	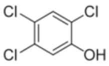	2.79±0.00	toxic in primary screen
19	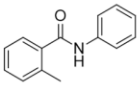	1.50±0.05	inactive (100 µM)
20	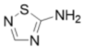	2.81±0.32	toxic in primary screen
21	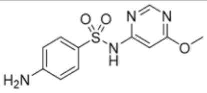	0.48±0.05	IC_{50} 71±32 (0.25±0.12 rem. act.)
22	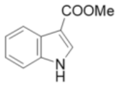	0.54±0.06	IC_{50} 48±11 (0.31±0.08 rem. act.)
23	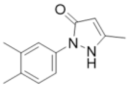	0.38±0.02	inactive (30 µM) toxic (≥ 50 µM)
24	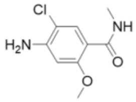	0.44±0.03	IC_{50} 54±11 (0.18±0.06 rem. act.)

b

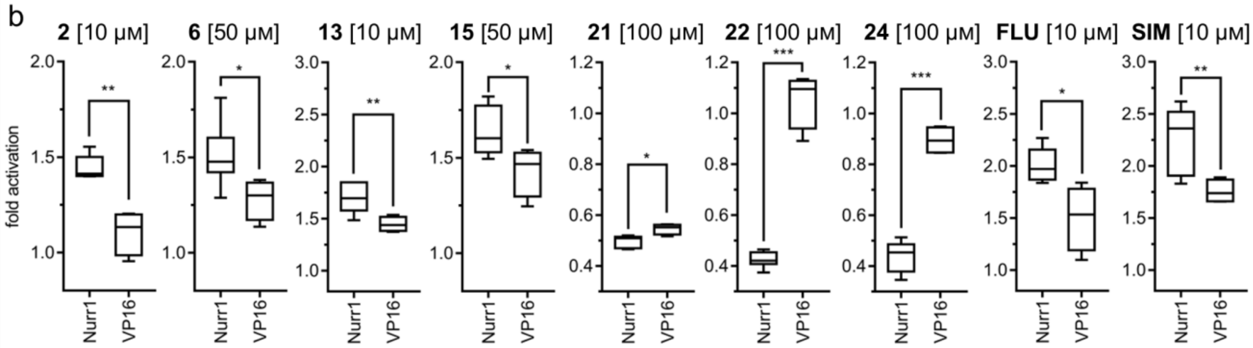

Figure S2. Follow up of the primary drug fragment screen for Nurr1 modulation. (A) 24 primary screening hits further considered and their Nurr1 modulatory activity in a Gal4-Nurr1 hybrid reporter gene assay. Reporter activity in primary screen is mean±SD reporter activity, n=2. Nurr1 modulation: only activities validated against Gal4-VP16 are reported. Data for fragment **22** from [1].EC_{50} or IC_{50} values are mean±S.E.M.; n≥3. Maximum activation or remaining activity refers to the maximum reporter activation or repression efficacy compared to DMSO (0.1%) treated cells. Toxic false positive hits from the initial screen were not further investigated. (B) Control experiments employing a Gal4-VP16 hybrid receptor were performed to confirm or refute Gal4-Nurr1 mediated activity in the cellular hybrid reporter gene assay. Boxplots show: center line, median; box limits, upper and lower quartiles; whiskers, min/max; $n \geq 4$. * $p < 0.05$, ** $p < 0.01$ *** $p < 0.001$ (t-test).

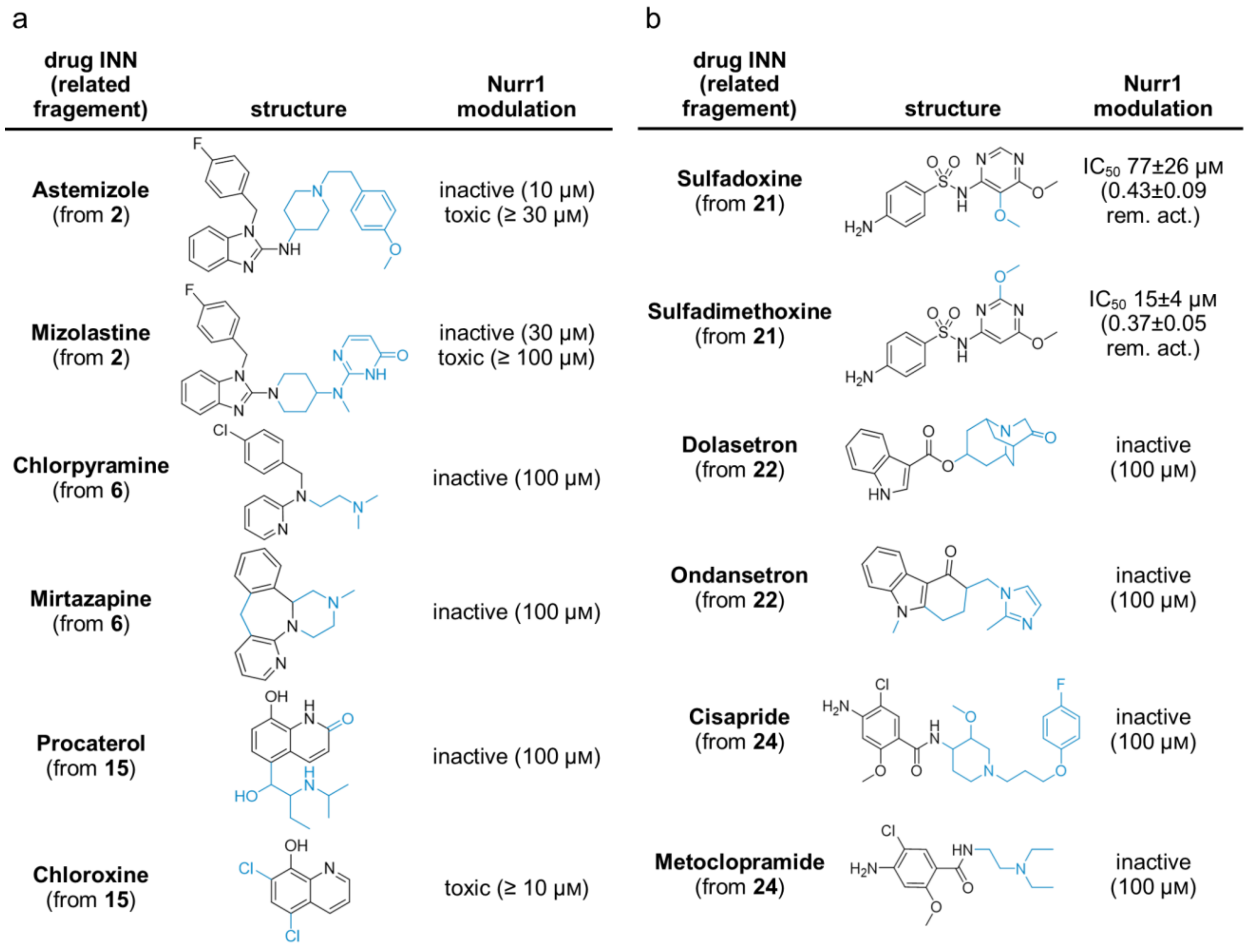

Figure S3. Nurr1 modulatory activity of fragment derived drugs. (a,b) FDA approved drugs related to validated Nurr1 activating (a) and repressing (b) fragments were tested on Nurr1 in a Gal4-Nurr1 hybrid reporter gene assay. Structural extensions compared to the underlying fragments are shown in blue. Only statins and the antibiotics sulfadoxine and sulfadimethoxine retained the Nurr1 modulatory activity of their fragment precursors **13** and **21** (see also Figure 1b). IC_{50} values are mean±S.E.M.; n≥3.

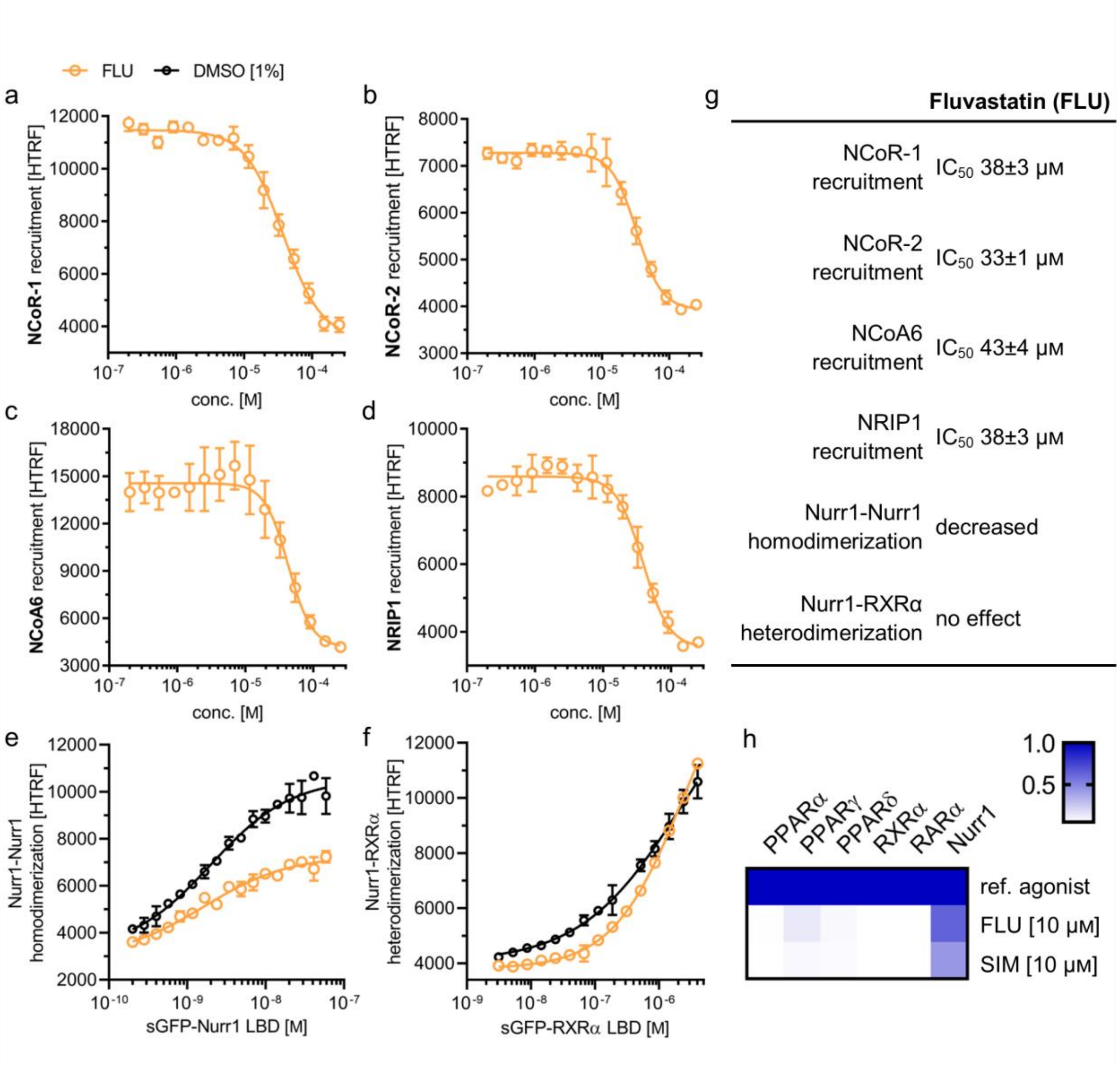

Figure S4. Effects of fluvastatin (FLU) on co-regulator interactions and dimerization of Nurr1. (a-d) Fluvastatin displaced NCoR-1 (a), NCoR-2 (b), NCoA6 (c) and NRIP1 (d) from the Nurr1 LBD. (e, f) Fluvastatin (30 µM) decreased homodimerization of Nurr1 (e) without affecting Nurr1-RXRα heterodimerization (f). Data are the mean±SD; N=3. (g) Summarized cell-free Nurr1 modulatory activities of FLU. (h) Selectivity profiles of FLU and SIM at 10 µM on related lipid-activated transcription factors in Gal4 hybrid reporter gene assays. Heatmap shows mean relative activation compared to reference agonists at 1 µM for PPARs (α: GW7647; γ: rosiglitazone; δ: L165,041), RXRα (bexarotene), RARα (tretinoin) and 100 µM for Nurr1 (amodiaquine); n ≥ 2.

Table S1. Effects of Simvastatin on differential gene expression in T98G cells. Only protein coding genes with log_2fold change > |0.5| that were selectively altered by Simvastatin in nt siRNA treated cells but not altered in Nurr1 silenced cells are shown.

Gene name	Gene description	log_2 (fold change)	$-log_{10}$ (p value)
**ARHGAP15**	Rho GTPase activating protein 15	4.807	2.186
**C9orf152**	chromosome 9 open reading frame 152	4.645	1.958
**CPA1**	carboxypeptidase A1	4.474	1.577
**FAM83F**	family with sequence similarity 83 member F	4.461	1.694
**CDH16**	cadherin 16	4.278	1.562
**RAB39B**	RAB39B, member RAS oncogene family	3.406	1.764
**CRHBP**	corticotropin releasing hormone binding protein	3.137	1.432
**TREM1**	triggering receptor expressed on myeloid cells 1	2.908	2.069
**TNF**	tumor necrosis factor	2.691	1.315
**NKX2-3**	NK2 homeobox 3	2.690	1.653
**BDKRB1**	bradykinin receptor B1	2.048	2.227
**TUBA8**	tubulin alpha 8	1.699	1.324
**FNDC11**	fibronectin type III domain containing 11	1.698	2.718
**IL4I1**	interleukin 4 induced 1	1.404	1.449
**PPM1J**	protein phosphatase, Mg2+/Mn2+ dependent 1J	1.353	1.321
**SERPINE1**	serpin family E member 1	1.312	15.575
**CXCL8**	C-X-C motif chemokine ligand 8	1.235	1.890
**INPP5D**	inositol polyphosphate-5-phosphatase D	1.223	1.567
**SIX2**	SIX homeobox 2	1.174	2.487
**BDKRB2**	bradykinin receptor B2	1.133	6.578
**TSPAN19**	tetraspanin 19	1.094	1.991
**ICAM5**	intercellular adhesion molecule 5	1.082	2.618
**DLG2**	discs large MAGUK scaffold protein 2	1.005	1.331
**P2RY8**	P2Y receptor family member 8	0.976	1.383
**TCAF2**	TRPM8 channel associated factor 2	0.944	3.036
**TNFSF14**	TNF superfamily member 14	0.932	1.959
**CES1**	carboxylesterase 1	0.900	2.513
**HSD11B1**	hydroxysteroid 11-beta dehydrogenase 1	0.835	1.724
**UBB**	ubiquitin B	0.825	41.528
**ANKRD37**	ankyrin repeat domain 37	0.821	3.810
**TMEM191C**	transmembrane protein 191C	0.791	1.388
**SCO2**	SCO2, cytochrome c oxidase assembly protein	0.791	1.338
**SLC6A9**	solute carrier family 6 member 9	0.767	30.239
**G0S2**	G0/G1 switch 2	0.741	2.227
**TRIM36**	tripartite motif containing 36	0.738	4.474
**CIART**	circadian associated repressor of transcription	0.727	5.046
**FYN**	FYN proto-oncogene, Src family tyrosine kinase	0.704	2.934
**CHAC1**	ChaC glutathione specific gamma-glutamylcyclotransferase 1	0.697	2.637
**TRIB3**	tribbles pseudokinase 3	0.688	21.592
**SEMA6A**	semaphorin 6A	0.685	2.272
**GDF15**	growth differentiation factor 15	0.683	3.861
**HAP1**	huntingtin associated protein 1	0.679	2.363
**EFNA3**	ephrin A3	0.658	1.372
**PECAM1**	platelet and endothelial cell adhesion molecule 1	0.655	2.512
**ABCC6**	ATP binding cassette subfamily C member 6	0.643	1.885
**PRR19**	proline rich 19	0.624	1.558
**PPP2R2C**	protein phosphatase 2 regulatory subunit Bgamma	0.623	1.630
**SERPINB8**	serpin family B member 8	0.623	5.933
**CD70**	CD70 molecule	0.597	2.813
**NPIPB3**	nuclear pore complex interacting protein family member B3	0.593	3.099
**GLI1**	GLI family zinc finger 1	0.583	1.324
**ZFYVE28**	zinc finger FYVE-type containing 28	0.574	3.045
**PAK6**	p21 (RAC1) activated kinase 6	0.572	1.971
**CXCR4**	C-X-C motif chemokine receptor 4	0.570	1.612
**KRT15**	keratin 15	0.569	1.447
**NFKBIZ**	NFKB inhibitor zeta	0.560	1.709
**PHLDA2**	pleckstrin homology like domain family A member 2	0.548	13.467
**PALM3**	paralemmin 3	0.542	1.432
**RILP**	Rab interacting lysosomal protein	0.536	2.972
**CHST2**	carbohydrate sulfotransferase 2	0.535	4.291
**GLIS3**	GLIS family zinc finger 3	0.532	2.027
**XKRX**	XK related X-linked	0.523	1.323
**RELB**	RELB proto-oncogene, NF-kB subunit	0.517	2.137
**HTR7**	5-hydroxytryptamine receptor 7	0.510	4.288
**EN2**	engrailed homeobox 2	0.507	1.775

**SH2D5**	SH2 domain containing 5	0.505	1.920
**XKR8**	XK related 8	0.503	1.467
**SLCO4A1**	solute carrier organic anion transporter family member 4A1	0.500	9.207
**GLRB**	glycine receptor beta	-0.502	1.449
**GPRASP1**	G protein-coupled receptor associated sorting protein 1	-0.504	1.683
**NALCN**	sodium leak channel, non-selective	-0.511	2.536
**SYDE2**	synapse defective Rho GTPase homolog 2	-0.516	1.762
**RASD2**	RASD family member 2	-0.518	1.486
**KCNB1**	potassium voltage-gated channel subfamily B member 1	-0.518	2.445
**RNFT2**	ring finger protein, transmembrane 2	-0.530	1.972
**MID2**	midline 2	-0.535	1.577
**C12orf60**	chromosome 12 open reading frame 60	-0.538	1.632
**GLT8D2**	glycosyltransferase 8 domain containing 2	-0.540	3.153
**PAPPA**	pappalysin 1	-0.541	1.788
**LAMA5**	laminin subunit alpha 5	-0.550	2.384
**SSPO**	SCO-spondin	-0.552	2.682
**HLA-DMB**	major histocompatibility complex, class II, DM beta	-0.553	1.712
**GOLGA8A**	golgin A8 family member A	-0.555	2.862
**GDAP1**	ganglioside induced differentiation associated protein 1	-0.557	4.551
**NAIP**	NLR family apoptosis inhibitory protein	-0.569	1.417
**IFI6**	interferon alpha inducible protein 6	-0.573	1.667
**TSSK4**	testis specific serine kinase 4	-0.574	1.482
**RNF152**	ring finger protein 152	-0.578	4.476
**MBLAC1**	metallo-beta-lactamase domain containing 1	-0.581	1.563
**GUCY1A1**	guanylate cyclase 1 soluble subunit alpha 1	-0.581	2.684
**CBLN2**	cerebellin 2 precursor	-0.595	3.691
**C2orf16**	chromosome 2 open reading frame 16	-0.606	1.700
**HMCN1**	hemicentin 1	-0.608	1.337
**PGM5**	phosphoglucomutase 5	-0.623	1.412
**ZFPM2**	zinc finger protein, FOG family member 2	-0.627	2.786
**HSF2BP**	heat shock transcription factor 2 binding protein	-0.628	1.310
**NOXA1**	NADPH oxidase activator 1	-0.628	1.325
**PPL**	periplakin	-0.632	2.024
**TTN**	titin	-0.633	1.994
**PPP2R2B**	protein phosphatase 2 regulatory subunit Bbeta	-0.636	2.072
**JCAD**	junctional cadherin 5 associated	-0.641	3.649
**METTL27**	methyltransferase like 27	-0.645	2.781
**EPHA4**	EPH receptor A4	-0.652	3.306
**GLI2**	GLI family zinc finger 2	-0.654	1.749
**KRT80**	keratin 80	-0.656	3.328
**KIT**	KIT proto-oncogene receptor tyrosine kinase	-0.659	1.559
**EFCAB6**	EF-hand calcium binding domain 6	-0.673	2.391
**ATOH8**	atonal bHLH transcription factor 8	-0.683	1.423
**ZNF154**	zinc finger protein 154	-0.701	1.459
**CSPG4**	chondroitin sulfate proteoglycan 4	-0.709	1.893
**COLGALT2**	collagen beta(1-O)galactosyltransferase 2	-0.726	1.373
**ADAMTS3**	ADAM metallopeptidase with thrombospondin type 1 motif 3	-0.733	1.873
**PDE5A**	phosphodiesterase 5A	-0.744	4.560
**GPT**	glutamic--pyruvic transaminase	-0.802	4.589
**EGR3**	early growth response 3	-0.809	1.421
**C1QTNF2**	C1q and TNF related 2	-0.814	1.623
**MOV10L1**	Mov10 like RISC complex RNA helicase 1	-0.830	3.639
**ZSCAN23**	zinc finger and SCAN domain containing 23	-0.832	1.536
**EPB41L4B**	erythrocyte membrane protein band 4.1 like 4B	-0.839	1.364
**GOLGA8N**	golgin A8 family member N	-0.840	2.326
**ABI3BP**	ABI family member 3 binding protein	-0.854	1.481
**CTTNBP2**	cortactin binding protein 2	-0.893	6.170
**TJP2**	tight junction protein 2	-0.919	1.606
**IGSF9B**	immunoglobulin superfamily member 9B	-0.970	4.234
**EGR2**	early growth response 2	-0.979	1.452
**NAP1L2**	nucleosome assembly protein 1 like 2	-1.015	1.472
**CRISPLD1**	cysteine rich secretory protein LCCL domain containing 1	-1.022	2.115
**TUBA3D**	tubulin alpha 3d	-1.038	1.757
**C4A**	complement C4A (Rodgers blood group)	-1.038	2.369
**AC092718.8**	novel protein	-1.144	1.533
**ENTPD8**	ectonucleoside triphosphate diphosphohydrolase 8	-1.271	2.076
**SLC4A4**	solute carrier family 4 member 4	-1.301	1.434
**POSTN**	periostin	-1.302	2.312
**ALPK3**	alpha kinase 3	-1.342	1.640
**PI15**	peptidase inhibitor 15	-1.348	4.241
**AC119396.1**	novel protein	-1.357	1.321

**CFAP57**	cilia and flagella associated protein 57	-1.399	1.433
**SPANXD**	SPANX family member D	-1.423	1.374
**PTGER2**	prostaglandin E receptor 2	-1.429	2.793
**GALNTL6**	polypeptide N-acetylgalactosaminyltransferase like 6	-1.431	1.511
**NTN3**	netrin 3	-1.442	1.443
**AC015802.6**	novel protein	-1.470	1.726
**AC233992.2**	novel transcript	-1.551	1.493
**XDH**	xanthine dehydrogenase	-1.578	1.482
**TEX52**	testis expressed 52	-1.581	2.121
**IFI27**	interferon alpha inducible protein 27	-1.641	2.426
**SPATA9**	spermatogenesis associated 9	-1.697	1.782
**NUDT7**	nudix hydrolase 7	-1.699	2.222
**RNF150**	ring finger protein 150	-1.739	3.840
**CXCR6**	C-X-C motif chemokine receptor 6	-1.770	1.586
**METTL7B**	methyltransferase like 7B	-1.799	1.363
**FSTL4**	follistatin like 4	-1.838	1.364
**FBLN2**	fibulin 2	-1.887	1.400
**ZPLD1**	zona pellucida like domain containing 1	-1.905	1.452
**CYFIP2**	cytoplasmic FMR1 interacting protein 2	-2.061	1.390
**APELA**	apelin receptor early endogenous ligand	-2.148	1.767
**ACMSD**	aminocarboxymuconate semialdehyde decarboxylase	-2.265	1.395
**IZUMO1**	izumo sperm-egg fusion 1	-2.306	1.445
**STPG1**	sperm tail PG-rich repeat containing 1	-2.366	1.743
**MMP12**	matrix metallopeptidase 12	-2.698	1.331
**EGR1**	early growth response 1	-2.825	3.705
**NFE4**	nuclear factor, erythroid 4	-3.019	1.366
**SENP3-EIF4A1**	SENP3-EIF4A1 readthrough (NMD candidate)	-3.614	1.948
**LRRC43**	leucine rich repeat containing 43	-3.615	1.424
**ATP6V1G3**	ATPase H+ transporting V1 subunit G3	-3.731	1.529
**TBC1D3K**	TBC1 domain family member 3K	-3.818	1.391
**AL136531.2**	novel transcript	-3.828	1.625
**LPAR5**	lysophosphatidic acid receptor 5	-3.888	1.743
**KLRC4-KLRK1**	KLRC4-KLRK1 readthrough	-3.888	1.740
**ACTG2**	actin, gamma 2, smooth muscle, enteric	-3.989	1.792
**ASPDH**	aspartate dehydrogenase domain containing	-4.067	1.390
**ABCB1**	ATP binding cassette subfamily B member 1	-4.091	1.428
**AL358472.6**	novel protein	-4.101	1.381
**KLRC4**	killer cell lectin like receptor C4	-4.469	1.860
**FAM71D**	family with sequence similarity 71 member D	-4.642	1.949
**TMEM179**	transmembrane protein 179	-4.899	2.096

Table S2. Effects of Simvastatin on differential gene expression in LPS-stimulated T98G cells. Only protein coding genes with log_2fold change > |0.5| that were selectively altered by Simvastatin in nt siRNA treated cells but not altered in Nurr1 silenced cells are shown.

Gene name	Gene description	log_2 (fold change)	$-log_{10}$ (p value)
**HK3**	hexokinase 3	4.559	2.002
**GJB5**	gap junction protein beta 5	4.250	1.605
**C3orf67**	chromosome 3 open reading frame 67	4.242	2.106
**LRRD1**	leucine rich repeats and death domain containing 1	4.195	2.135
**CLEC18A**	C-type lectin domain family 18 member A	4.083	1.379
**CFHR1**	complement factor H related 1	4.083	1.439
**KLHL35**	kelch like family member 35	4.080	1.397
**COL9A2**	collagen type IX alpha 2 chain	4.070	1.368
**RNF43**	ring finger protein 43	4.004	1.785
**RNF222**	ring finger protein 222	3.708	1.545
**C16orf46**	chromosome 16 open reading frame 46	3.367	1.431
**MUC15**	mucin 15, cell surface associated	2.549	1.302
**RCAN2**	regulator of calcineurin 2	1.823	2.312
**ATP10B**	ATPase phospholipid transporting 10B (putative)	1.761	1.744
**DNAJB13**	DnaJ heat shock protein family (Hsp40) member B13	1.752	1.835
**C11orf96**	chromosome 11 open reading frame 96	1.704	3.066
**PHOSPHO1**	phosphoethanolamine/phosphocholine phosphatase	1.339	1.303
**SNAI3**	snail family transcriptional repressor 3	1.124	3.420
**TMEM216**	transmembrane protein 216	1.116	2.046
**NOTCH4**	notch 4	1.020	1.821
**EPS8L1**	EPS8 like 1	1.019	1.896
**C19orf73**	chromosome 19 open reading frame 73	0.842	1.828
**AKAP12**	A-kinase anchoring protein 12	0.837	1.637
**XKRX**	XK related X-linked	0.831	2.609
**CCDC151**	coiled-coil domain containing 151	0.816	1.625
**PTGS2**	prostaglandin-endoperoxide synthase 2	0.789	8.047
**FKBP1B**	FK506 binding protein 1B	0.781	1.336
**DNAAF3**	dynein axonemal assembly factor 3	0.778	2.180
**INHBE**	inhibin subunit beta E	0.771	1.551
**CPEB3**	cytoplasmic polyadenylation element binding protein 3	0.749	1.484
**HSD11B1**	hydroxysteroid 11-beta dehydrogenase 1	0.738	1.525
**CD7**	CD7 molecule	0.713	2.130
**SLC2A5**	solute carrier family 2 member 5	0.688	1.390
**C4A**	complement C4A (Rodgers blood group)	0.685	1.540
**ABCA3**	ATP binding cassette subfamily A member 3	0.680	1.863
**ICAM1**	intercellular adhesion molecule 1	0.675	4.838
**STBD1**	starch binding domain 1	0.640	1.932
**EN2**	engrailed homeobox 2	0.638	3.077
**SWSAP1**	SWIM-type zinc finger 7 associated protein 1	0.632	1.534
**CDKN2D**	cyclin dependent kinase inhibitor 2D	0.631	6.371
**ATP6V0C**	ATPase H+ transporting V0 subunit c	0.618	2.651
**TMPRSS6**	transmembrane serine protease 6	0.610	1.352
**PAK6**	p21 (RAC1) activated kinase 6	0.602	1.844
**TUBB2A**	tubulin beta 2A class IIa	0.600	6.127
**SERPINE1**	serpin family E member 1	0.599	4.268
**MT1X**	metallothionein 1X	0.547	4.060
**TRNP1**	TMF1-regulated nuclear protein 1	0.542	2.065
**ETV4**	ETS variant 4	0.538	12.089
**SEMA6A**	semaphorin 6A	0.512	1.731
**CBLN2**	cerebellin 2 precursor	-0.502	2.847
**DENND2A**	DENN domain containing 2A	-0.506	1.386
**KIAA0319**	KIAA0319	-0.515	1.774

**TLN2**	talin 2	-0.516	4.222
**IL31RA**	interleukin 31 receptor A	-0.517	3.058
**LEAP2**	liver enriched antimicrobial peptide 2	-0.526	1.638
**LDLRAD4**	low density lipoprotein receptor class A domain containing 4	-0.529	1.761
**S100A1**	S100 calcium binding protein A1	-0.539	1.517
**GABRA3**	gamma-aminobutyric acid type A receptor alpha3 subunit	-0.555	2.330
**GATA2**	GATA binding protein 2	-0.559	1.589
**KCNB1**	potassium voltage-gated channel subfamily B member 1	-0.571	3.349
**TRPV2**	transient receptor potential cation channel subfamily V member 2	-0.580	1.503
**PSG4**	pregnancy specific beta-1-glycoprotein 4	-0.588	1.799
**NPIPB9**	nuclear pore complex interacting protein family member B9	-0.616	1.306
**ZNF835**	zinc finger protein 835	-0.617	1.418
**ANKRD23**	ankyrin repeat domain 23	-0.640	1.324
**ZNF10**	zinc finger protein 10	-0.641	2.733
**CKMT2**	creatine kinase, mitochondrial 2	-0.729	1.788
**TNXB**	tenascin XB	-0.741	1.565
**MYH3**	myosin heavy chain 3	-0.772	1.930
**FGF14**	fibroblast growth factor 14	-0.789	1.989
**COX16**	cytochrome c oxidase assembly factor COX16	-0.808	1.951
**PI15**	peptidase inhibitor 15	-0.811	2.124
**TSTD3**	thiosulfate sulfurtransferase like domain containing 3	-0.832	1.303
**AKAP5**	A-kinase anchoring protein 5	-0.847	1.468
**SLC16A14**	solute carrier family 16 member 14	-0.855	1.412
**FHOD3**	formin homology 2 domain containing 3	-0.860	1.560
**AC009336.2**	homeobox D4	-0.918	1.433
**CDRT15**	CMT1A duplicated region transcript 15	-1.043	1.479
**KCNA7**	potassium voltage-gated channel subfamily A member 7	-1.093	1.686
**TYRP1**	tyrosinase related protein 1	-1.165	2.589
**FBXO24**	F-box protein 24	-1.201	1.965
**DNAH7**	dynein axonemal heavy chain 7	-1.204	1.679
**CPA5**	carboxypeptidase A5	-1.210	1.618
**MARCH4**	membrane associated ring-CH-type finger 4	-1.219	1.886
**SLC35G6**	solute carrier family 35 member G6	-1.249	1.402
**C3orf80**	chromosome 3 open reading frame 80	-1.420	1.404
**PROSER2**	proline and serine rich 2	-1.728	2.285
**PDE6B**	phosphodiesterase 6B	-1.752	1.471
**METTL7B**	methyltransferase like 7B	-1.838	1.388
**BEGAIN**	brain enriched guanylate kinase associated	-1.841	2.021
**GRM4**	glutamate metabotropic receptor 4	-2.086	1.341
**RAB11FIP1**	RAB11 family interacting protein 1	-2.102	1.743
**APELA**	apelin receptor early endogenous ligand	-2.210	1.466
**DEF6**	DEF6, guanine nucleotide exchange factor	-2.264	2.100
**NANOS3**	nanos C2HC-type zinc finger 3	-2.471	1.420
**MYBPC1**	myosin binding protein C, slow type	-2.520	1.808
**ACSL6**	acyl-CoA synthetase long chain family member 6	-2.891	1.999
**ANKRD65**	ankyrin repeat domain 65	-3.200	1.496
**GPR83**	G protein-coupled receptor 83	-3.808	1.638
**DEGS2**	delta 4-desaturase, sphingolipid 2	-3.882	1.673
**SLC6A12**	solute carrier family 6 member 12	-4.119	1.445
**VSTM5**	V-set and transmembrane domain containing 5	-4.238	1.512
**HORMAD1**	HORMA domain containing 1	-4.282	1.606
**GSDMC**	gasdermin C	-4.357	1.454
**SLC35F1**	solute carrier family 35 member F1	-4.426	2.395
**EBF3**	early B cell factor 3	-4.531	1.889
**HS6ST2**	heparan sulfate 6-O-sulfotransferase 2	-4.656	2.089
**ZNF366**	zinc finger protein 366	-4.718	2.046
**TBC1D3B**	TBC1 domain family member 3B	-7.221	2.770

Supporting Methods

Computational Methods.

General: Calculations were conducted in KNIME (version 3.7.2, KNIME AG, Zurich, Switzerland) and Molecular Operating Environment (MOE, version 2018.0101, Chemical Computing Group Inc. Montreal, QC, Canada) using default settings for each tool/function unless stated otherwise. Amber10:EHT was used as default force field for all calculations. *Library processing*: Analysis of the drug fragment library from Prestwick (Prestwick Chemical, Illkirch, France) was performed in KNIME using the provided SMILES strings compared to the DrugBank database (all drug structures in SDF Format, version 5.1.1, released on 2018-07-03). The RDKit extension nodes (version 4.0.1.v202002121354) were used to filter for PAINs structures and calculate features (MW, clogP, number of H-bond donors/acceptors and rotatable bonds, aromatic rings, TPSA). For hits from the primary screen, a search for parent and related drugs was performed via molecule substructure and murcko scaffold (both RDKit nodes) compared to the DrugBank database (version 5.1.1). Graph based frameworks were extracted with the MOE KNIME extension node murcko frameworks ignoring small terminal rings of size 3 or 4. Rings of size 5 to 7 atoms as well as annealed rings, bicyclo and spiro compounds of equal ring count were assigned to the same groups. Geometry in terms of linker attachment points and connectivity was ignored, only the linker length was considered. *Multiple alignment*: Molecular structures of amodiaquine, fluvastatin and pitavastatin were prepared using MOE Wash tool: protonation state dominant at pH 7; coordinates rebuild 3D; preserved existing chirality. Multiple alignment of these three compounds was performed using default settings from MOE flexible alignment tool.

Supporting References

[1] D. Zaienne, S. Willems, S. Schierle, J. Heering, D. Merk, *J. Med. Chem.* **2021**, *64*, 15126–15140.
